# COVID-19 vaccination and governance in the case of low, middle and high-income countries

**DOI:** 10.1186/s12889-023-15975-3

**Published:** 2023-06-05

**Authors:** Dan Lupu, Ramona Tiganasu

**Affiliations:** 1 Faculty of Economics and Business Administration, Alexandru Ioan Cuza University of Iasi, Carol I Boulevard, No. 22, Iasi, Romania; 2 Faculty of Law, Centre for European Studies, Alexandru Ioan Cuza University of Iasi, Carol I Boulevard, No. 19, Iasi, Romania

**Keywords:** COVID-19 health crisis, COVID-19 vaccination, Governance, Low, middle and high-income countries, Public policies

## Abstract

**Background:**

Global crises, regardless of the place where they started to spread or of the factors that triggered them, require a comprehensive approach, primarily based on good communication, cooperation and mutual support. No individual and no institution should remain indifferent to crises but, on the contrary, be fully aware that any involvement in curbing them matters. Although humanity can be affected by various types of crises, in this paper we refer to the one related to COVID-19 pandemic. There are certain reasons that come to justify our choice: first of all, being a shock with a strong impact on people, its analysis should be performed from several angles; this may bring to light an image with its disparate propagation and measures to counteract it both in developed countries, and especially in those with a shortage of resources. Secondly, in the context of the emergence of vaccines against COVID-19, it is helpful to have an overview of COVID-19 through the lens of the relationship between the vaccination process and the elements that characterize governance, with a differentiated dashboard by country categories worldwide: low, middle and high-income countries. Our study is far from capturing the complexity arising from such social problem, but rather aims to outline the defining role of governance when it comes to providing firm reactions to the COVID-19 crisis.

**Methods:**

Given that our sample consists of a large number of countries, namely 170, first, examined all together, and then, split into three groups (high, middle and low-income), it is challenging to address governance in association with COVID-19 vaccination, in order to see how much they interact and how each of the six aggregate governance indicators of the World Bank (Worldwide Governance Indicators) is reflected in this process. Even if they do not oscillate strongly over relatively short periods of time, reporting on health issues requires a sequential inventory, considering closer time intervals, so as to be able to act promptly. Thus, to better distinguish how the COVID-19 vaccination process evolved in low, middle and high-income countries, but also how it was imprinted by governance, we present the situation quarterly (March, June, September and December), in 2021, the year when the immunization campaigns were the most intense at the global level. Regarding the applied methods, we mention both OLS regressions with robust estimators and a panel model, used to investigate the determinants of COVID-19 vaccination, some of them describing the good governance, as well as other dimensions.

**Results:**

The findings point out that the influence of governance on COVID-19 vaccination differs depending on whether a country belongs to high, middle or low-income typology: the strongest determinism of governance on vaccination is encountered in high-income countries, and the weakest in low-income ones; in some cases, governance does not matter significantly. However, exploring the three groups of states included in the research, it is observed that the most relevant factors in this relationship are government effectiveness, regulatory quality and control of corruption.

**Conclusions:**

Besides the order of importance of governance indicators on COVID-19 vaccination, our study indicates that, overall, governance positively shapes the vaccination rate at the level of the chosen sample. In normative terms, these findings can be translated particularly by the fact that they can serve as information to raise awareness on the relevance of the existence of an institutional framework that allows the formulation of strategies according to the patterns of each country, especially since the actionable tools depend on the available resources. As a general conclusion, public policies should be designed in such a way as to strengthen trust in vaccination regulations and in governments, to reduce the multifaceted negative effects of this health crisis and to hope for its total end.

**Supplementary Information:**

The online version contains supplementary material available at 10.1186/s12889-023-15975-3.

## Introduction

The exploration of COVID-19 vaccination is a topic of scientific interest, especially since the possibility to completely overcome the pandemic lies precisely in this desideratum. In order to have an overview of the virus transmissibility and pathogenicity, the statistics provided by [1, 2] are eloquent: COVID-19 indicators have registered strong variations worldwide, gathering more than 290 million cases and 5.45 million deaths until January 1, 2022. Thus, facing enormous humanitarian and economic losses, national governments, international organizations and scientists have made substantial efforts to develop effective treatments and vaccines against the disease [3, 4]. Accordingly, during 2020, several types of vaccines were approved, and, subsequently, produced on a large scale and distributed internationally.

Vaccines are one of the most successful discoveries in the history of mankind and medicine. Over the course of time, global vaccination programmes have saved many lives by strengthening the immune system against pathogens and ensuring individual health protection [5, 6]. Without the promotion of such vaccination programmes, public health and economy would have been seriously affected [7, 8]. By immunizing a high percentage of the people, mass immunization can be obtained, which favours the limitation of virus diffusion of any type, offering, at the same time, protection for unvaccinated or immunocompromised individuals [9]. Unlike natural immunization, which can have multiple serious consequences, immunization by vaccination has been shown to play a major role in providing a response to pandemic eradication, becoming clear that there is a direct link between the administration of various vaccines and a contraction in the mortality rate [10, 11]. World Health Organization (WHO) has set a threshold to achieve mass vaccination in the COVID-19 case, this being around 70%; reaching this threshold is a tool for limiting the disease, but also a big challenge for the health system and national governments [12, 13]. The countries have adapted to the epidemiological evolution and, although most of the COVID-19 prevention and control measures were relatively similar, their implementation was different [14–18]. Some states applied tougher restrictions, others limited themselves to imposing rather basic recommendations; nevertheless, regardless of the approach, the institutions are the ones that stamp the routes to be followed to overcome this crisis.

Tackling coronavirus is, therefore, a major global concern, requiring a lot of financial and human resources to manage it properly [19–22]. In terms of vaccination, the discrepancies between countries obviously occur as a result of the influence of various factors. It is particularly for that reason that we chose this study to be dedicated to capturing the differences on COVID-19 vaccination on a sample of countries, from an income perspective. We used the World Bank country classifications by income, this typology being accepted internationally. The established thresholds for distinguishing the groups of countries are expressed in gross national income (GNI) per capita in current USD and are adjusted annually in relation to inflation. Thus, for the year 2021, the tresholds are: for high-income countries (> 12 695 GNI per capita in USD), upper-middle income (4096 – 12 695 GNI per capita), lower-middle income (1 046 – 4 095 GNI per capita), low-income (< 1 045 GNI per capita). There are strong social inequalities between these categories of states, which also attract differences in terms of purchasing vaccines. Practically, the impossibility of some vulnerable groups to have access to anti-COVID-19 vaccines highlights, especially in times of crisis, the incapacity of institutions to provide basic services and humanitarian aid through joint efforts [23]. Given that the right to life is universal, an approach by which states in difficulty in procuring vaccines be supported should be found. By reporting to the level of development of a country, some studies have shown that gross domestic product (GDP) per capita influences the COVID-19 spread and vaccination of the population [24–29].

Putting vaccination in connection with governance finds its explanations particularly on the background of the need to have reliable information based on which the most appropriate decisions can be made, a central role on this line being played by national governments [30, 31]. Also, the conditionalities between the quality of institutions and the acceptance of vaccination should be considered [32–34]. As a consequence, ensuring good governance during crises is not an easy task, especially if states do not have adequate resources to direct towards the most affected sectors [35]. At the global level, it was found that, in the fight against COVID-19, the ability of governments to react promptly and implement measures according to the national specificity, as well as the degree of trust that citizens have in decision-making institutions, have a special relevance [36].

Informal institutions, which have permeated the collective mind for decades, largely shape the type of crisis response [37]. If the historical patterns of European states are taken into account, the East–West axes in institutional, economic, social terms can be easily distinguished and this is partly explained by the influence of the path dependence process. What is difficult to change over many decades translates in an inertia and an institutional maladaptation, which stand in the way of innovative crisis response mechanisms. Thus, in relation to the current health crisis, it is imperative that specialized institutions ensure effective communication with citizens, and that governments have an objective approach towards vaccination, conducting information campaigns to increase public confidence in the effectiveness of the vaccine, based on scientific evidence [38]. However, the strengthening of governance cannot be achieved in a very short period of time, but this should be a desideratum because, generally, the societies that enjoy a productive management from the actors with decision-making power are able to have adequate answers to the threats that arise.

All countries suffered losses due to COVID-19, but those with stronger governance and institutions were more likely to cope better with it [39, 40]. Public health could be shaped by governance, both by designing and implementing policies in the field, and by mobilizing and coordinating citizens and public institutions to achieve certain objectives [14]. Starting from this assumption, we propose to capture a quarterly dashboard in terms of governance and COVID-19 vaccination relationship, related to the year 2021, on a sample consisting in 170 countries. There is a need for in-depth analyses to investigate the interdependencies between the specific governance indicators and the COVID-19 vaccination rate, with the establishment of an order of their importance in the process. Precisely, the study focuses on the COVID-19 vaccination in countries around the world and separately by groups of countries (high, middle and low-income) in relation to governance. The paper presents, subsequently, aspects that refer to the literature review, methods and data used in the analysis, the research results and the main conclusions and suggestions for increasing the efficiency of public health policies aimed at the COVID-19 vaccination process.

## Literature review

The provision of medical services in the public sector could be put in association with the quality of governance and institutions [15], and the influence mechanisms can target doctor-patient, hospital-patient or hospital-service provider relationships [5, 16]. Deficient governance does not remain without repercussions, including in the health field (e.g., poor quality of health services, irrational investments, lack of action tools in case of systemic crises, inability to anticipate dangers, decrease in trust in health institutions, etc.) [41]. Specifically, poor governance imprints the health care system both at the micro and macro levels: distorted public procurement, excessive costs towards unnecessary investments, unfairness in obtaining public positions [42]. Taken together, all these negative manifestations lead to a decline in performance, by undermining medical productivity, especially in less developed countries. Financial expenses in the public sector call for a detailed oversight to properly balance inputs and outputs [29, 43]. Moreover, in times of crisis, adequate management is required, which does not allow for even greater deviations in terms of wasting resources. According to [44], there are several categories of strategic planning policies for a health crisis, divided into phases: emergency preparedness, crisis management, response and recovery; the above-mentioned study [44], which is based on the evaluation of 18 OECD countries, including Belgium, Denmark, France, Italy, Lithuania, the Netherlands, Norway, Switzerland, Sweden, the United Kingdom, highlights, through a content analysis, that after the first waves of the pandemic, the preparation for dealing with it was broadly insufficient, mainly in relation to the number of deaths or people left with sequelae after coronavirus infection. Then, despite the measures taken to alleviate the crisis, efforts were made to reduce its negative social and economic effects, but the loss of well-being was felt in many cases [17]. Previous studies have shown that when national governments tend to adopt stricter measures to fight against COVID-19, the outcomes can be more meaningful [45–47]. Risks can be more easily counteracted through institutional transparency, open communication between stakeholders and closeness to citizens. In the context of such challenges, effective crisis management is decisive, and the ability to anticipate possible system disruptions, the speed of response, the coordinated action by institutions and cooperation make the difference between states in terms of the adverse implications of crises [9].

Considering that, in our study, the year of interest is 2021, in the following, we briefly present the main results of the European Quality of Government Index (EQI), and the European Commission’s Standard Eurobarometer 94 “The EU and the pandemic coronavirus”, both published in 2021, in order to have an overview of the essential aspects analyzed in the paper, reflected in the case of European economies, whose particularities we know better. EQI measures the perceptions of public sector corruption, the quality of public services and their impartial allocation, being developed under the auspices of the University of Gothenburg in Sweden, on a sample of 129 000 respondents from 208 regions in the 27 member states of the European Union (EU) [48]. Unlike previous editions, the 2021 index may also reflect the connection with the efficiency of European governments’ crisis management and the response policies to the COVID-19 pandemic. It can be noticed that the perceptions in relation to governance in the European regions of Western Europe were better compared to those in Southern and Eastern Europe, which strengthens the causality between the elements that are part of this index and the quality of the measures designed to alleviate the effects of the COVID-19. In addition, Standard Eurobarometer 94 (2021) captures, among other things, the attitude of EU citizens towards the vaccination against COVID-19 and the sources of information which they would trust [49]. Opinion polls were conducted through online or face-to-face interviews, with more than 1000 respondents in each EU country, with the exception of Cyprus (505 respondents), Luxembourg (599), and Malta (535). In total, 27 409 people were questioned. Regarding the confidence in the EU as an information player, especially on the role of vaccination and vaccine safety, this is relatively high, as 59% of respondents believe that the EU will act accordingly to future challenges related to the coronavirus. Furthermore, most respondents consider that a vaccine is the only way humanity can get rid of the pandemic: Denmark (89%), Finland (88%), Sweden (86%), Italy (83%), Ireland ( 81%), the Netherlands and Belgium (77%). Lower percentages are found in Cyprus (46%), Bulgaria and France (54%), Austria (59%), Latvia and Poland (62%), Romania and Lithuania (65%), with the EU27 mean of 70% [49]. Thus, in the mirror, the two reports (EQI 2021 and Standard Eurobarometer 94) emphasize a centre-periphery pattern for the EU’s economies (East–West division), in the sense that a higher quality of institutions (West) leads to scale effects regarding the vaccination rate, as well.

By referring to the above aspects, in the specialized literature, several key points are emphasized. For example, since 2002, [5] have analyzed the factors that influence vaccination and demonstrated that the political environment and the contact with international agencies are essential. In addition, the effect of corruption on the vaccination was brought to light in the case of the Philippines and it was found that this phenomenon has negative implications: reduced vaccination rates, delayed vaccination of children, increased waiting time for health services, more affected areas being rural and poor [41]. Regarding the vaccination of children, a research was carried out in 48 low and middle-income countries to identify the main influencing factors and it was highlighted that, in addition to a number of economic and social variables (GDP per capita, number of doctors, health expenditure), the institutional environment is also particularly relevant [42]. As it has been pointed out over time, children are protected from diseases such as measles, mumps, and whooping cough as a result of vaccines; consequently, in the context of the current pandemic, there is a need for effective communication with parents about the viability of vaccines, so that immunizations in this age group continue in a sustainable way [50]. COVID-19 vaccination has also been approached by [51] in a sample of 140 countries and it has been shown that there are significant differences, particularly caused by public corruption [52]. Analyzed the COVID-19 vaccination-related issues in the United States (US) and concluded that, especially in the early stages of this process, there were corrupt behaviours based mainly on the population’s desire to be immunized earlier than necessary. In another paper [53], the various socio-economic factors for COVID-19 vaccination in 50 US states were captured and the main finding was that where corruption is higher, lower vaccination rates are encountered.

During the vaccination process, many syncopes occurred internationally. For instance, the study of [4] emphasizes that of the 129 states that provided statistics on the progress of vaccination, more than half reported partial interruptions or even total suspension of this between March and April 2021; these were due to different issues related to the existence of unproper conditions for the storage of vaccines, disruptions of electronic record systems, inconsistency in the application of measures, certain delays in the supply of vaccines, etc. In addition to this research, the study of [36] analyzes 178 countries between March 20 and April 8, 2020 in order to determine the effectiveness of government policies in response to the crisis; according to this paper (p.1), “a one-unit increase in government response measures leads to a 0.353% upturn in public trust in the government and a 0.414% rise in its veracity”. To point out whether countries with a higher quality of governance are also those where the vaccination process is more efficient, [54] explored the situation at the level of 167 states, concluding that where there is low governance, more especially in poorer countries, supplementary aid is needed from various institutions to reduce the spread of the coronavirus.

Since the countries of the world experienced significant variations in terms of COVID-19 vaccination, notably in the period immediately following the appearance of vaccines, there were scientific concerns to see what the most important factors influencing vaccination were. Thus, by studying the results of the COVID-19 vaccination achieved until March 31, 2021, in the case of 128 countries, [55] established that the government effectiveness seen as the internal capacity of a state to manage a destabilizing situation is of great significance in the absorption of vaccines while its external capacity perceived as soft power is rather an element of robust conditionality. Besides, [56] carried out a study on 151 countries and revealed that, in the vaccination process, a special role is played by campaigns applied as widely as possible territorially but, although necessary, they are not sufficient to minimize the effects of the COVID-19 crisis in terms of mortality caused by the SARS-CoV2 virus; moreover, according to the mentioned author [56], in order to face future health threats, states should strengthen their public governance component, invest in new technologies, allocate greater resources to health and specialists from various fields to cooperate to develop an integrative and multidisciplinary strategy to combat pandemics. However, all these become problematic, particularly for low-income countries. Even if global public health strategies come with sets of actions that countries can follow to fade crises, a series of limitations intervene when it comes to their ability to effectively go through the entire chain of procedures related to the vaccination process, from procurement and regulations to distribution and absorption [57].

Even if little attention has been paid to the control policies of COVID-19 and the ethical issues associated with them, which also impede economic recovery [58, 59], there have been concerns related to the communication strategies for the application and distribution of vaccines so that immoral actions can be prevented as much as possible. Thus, a research applied in France, Germany, Sweden, the United Kingdom and Switzerland shows that an increase in public trust and a high absorption of vaccines can be achieved by providing scientific advice by those involved in the field, transparency and effective crisis management [60]. Moreover, the acceptance of the COVID-19 vaccine was correlated with elements that refer to age, place of origin, financial situation, level of education, existing medical conditions, mental well-being, government trust [61].

Governance’s responses to all these challenges, although varying from country to country, should be provided in a comprehensive manner, in the sense that solutions to a problem may require solving others [62, 63]. Therefore, public policies and governance need to work together to better identify cause-and-effect interactions and to anticipate their large-scale implications.

## Methods and data

The hypothesis from which we start is to emphasize whether in the COVID-19 vaccination process, the countries with good governance behave better than those with weaker institutions. To test it, we have applied a panel model, followed by checking the robustness of results using OLS regression and panel model with changed variables. According to the [64], by good governance is understood, in particular, the capacity of governments to develop and apply sound policies. By transposition to the main objective of our paper, that of seeing if governance influences the COVID-19 vaccination, good governance denotes the ability of governments to respond effectively to combat the diversity of issues that a pandemic induces, including the management of the vaccination process. To highlight this, six aggregate components of good governance under the umbrella of World Bank (control of corruption, government effectiveness, political stability, regulatory quality, rule of law and voice & accountability) were included in the analysis [64]. We chose to refer to these indicators because they cover a fairly broad sample of countries and, over time, they have served as a guide in designing public policies, being collected from multiple official data sources. Generally, governance indicators do not vary much from year to year, but in our research the emphasis falls on the presentation of the evolution of vaccination against COVID-19, which can be better captured if the data are presented on shorter periods, in our case, quarterly (March, June, September and December) and if it is considered that, in the COVID-19 vaccination regulations, frequent changes could appear. We chose to refer to the year 2021 because it was the year that followed the disasters recorded in terms of deaths due SARS-CoV-2 virus infection and also because, with the success of obtaining COVID-19 vaccines, since the end of 2020, it has been challenging to ascertain whether, starting from these realities, the COVID-19 vaccination rate experienced significant dynamics afterwards.

When discussing about COVID-19, the approach is usually oriented towards individuals, narrower samples and time-limited analyses. In order to cover and investigate the research topic as comprehensively as possible, an extensive data set was collected from various sources, for 170 countries around the world, framed in: high-income countries (53), middle-income countries (72) and low-income countries (45), defined according to the World Bank specifications, mentioned in the Introduction part. The sources of the variables, included in the study, are WHO, World Bank, Our World in Data/Worldometer, Oxford COVID-19 Government Response Tracker, with quarterly data for COVID-19 deaths and COVID-19 vaccination rate, and annual values for a series of macroeconomic and health control variables. The following general model is formulated:

1 $$COVID-19\ vaccination = f (Governance\ indicators: CC, GE, PS, RQ, RL, V\&A, Comorbidities, GDP\ per\ capita, Stringency, COVID-19\ deaths)$$

The above model can be transformed into a regression as shown in Eq. 2 below.

2 $$COVID-19\ {vaccination}_{t} = {\beta }_{0} + {\beta }_{1}COVID-19\ {vaccination}_{t} + {\beta }_{2}Governance\ {indicators}_{t} + {\beta }_{3}{Comorbidities}_{t} + {\beta }_{4}GDP\ per\ capita + {\beta }_{5}{Stringency}_{t} + {\beta }_{6}COVID-19\ {deaths}_{t}$$

Given that during the COVID-19 pandemic some reporting may have time delays, it is assumed that the regression model may, also, undergo adjustments, taking into account the values of the indicators at an earlier date (e.g., for COVID-19 vaccination, stringency, COVID-19 deaths). Incorporating lagged variables into the analysis could help overcome possible specification biases.

3 $$COVID-19\ {vaccination}_{t} = {\beta }_{0} + {\beta }_{1}COVID-19\ {vaccination}_{t-1} + {\beta }_{2}Governance\ {indicators}_{t} + {\beta }_{3}{Comorbidities}_{t} + {\beta }_{4}GDP\ per\ capita + {\beta }_{5}{Stringency}_{t-1} + {\beta }_{6}COVID-19\ {deaths}_{t-1}$$

where *COVID-19 vaccination* represents the percentage of the fully vaccinated population; governance is defined by the six indicators, which refers to the government’s ability to ensure a framework to prevent and combat corruption (*control of corruption: CC*), to formulate policies that provide quality public services (*government effectiveness: GE*), to generate stability and the absence of violence (*political stability: PS*), to put sound regulations into practice (*regulatory quality: RQ*), to respect the laws (*rule of law: RL*) and to be responsible, including in the relationship with its citizens (*voice & accountability: V&A*). The values for the Worldwide Governance Indicators (WGI) of World Bank range from -2.5 to + 2.5, but in our research the percentile rank (with values between 0–100) is used, higher values meaning better governance. *Comorbidities* are associated with chronic respiratory disease, diabetes, cardiovascular disease, cancer etc., all these making the human body even more vulnerable in the event of contracting a severe virus such as SARS-CoV2. The *GDP per capita*, expressed in purchasing power parity rates and divided by the total population, is included in the study to observe if countries with a higher GDP have a greater capacity to maneuver resources properly to combat the COVID-19. The *stringency index* designates the strictness of government responses towards COVID-19, ranging from 0 to 100, where 0 means no measure to fight the COVID-19. This indicator does not indicate the efficiency of the response of government policies, but rather degrades their strictness; the higher the stringency index, the stricter the government policies. *COVID-19 deaths* is the number of deaths caused by COVID-19 per 1 million population. To investigate the determinants of COVID-19 vaccination, in the second stage of the analysis, we will use a panel linear regression model. The equation of the panel type model is the same as equation no. 3, with the specification that fixed effects are tested.

## Research results

In the following, the main research results will be highlighted and the primary aspects that need to be considered in order to improve COVID-19 vaccination rate will be captured. Thus, Table 1 shows the main descriptive statistics of the indicators used for analysis. On average, until the end of December 2021, a vaccination rate of 49.257 was achieved at the level of all 170 states in the sample, with the highest percentages in United Arab Emirates, Cuba, Brunei, Portugal, Chile (over 90% of the population) and the lowest in Yemen, Chad, Haiti, Democratic Republic of Congo, Burundi (below 2%). Divided into the three groups of countries included in our study, it can be seen that the greatest rate of vaccination against COVID-19 was recorded in the case of those classified as high-income, reaching an average of 73.709, with a maximum of 98.990 (United Arab Emirates) and a minimum of 39.570 (Bahamas). For the other categories, the following results were displayed: for middle-income countries (the vaccination mean is 50.746, with a maximum of 92.330 for Cuba and a minimum of 2.960 for Cameroon), and for low-income countries, the vaccination mean is 16.540, with a maximum of 57.330 for Rwanda and a minimum of 0.040 for Burundi. Regarding the governance indicators, as a whole, the highest average is found at the RQ level (49.745), Singapore, New Zealand, Finland, Luxembourg, Australia (over 97) being ranked in the upper hierarchy, and Syria, Somalia, South Sudan, Venezuela, Libya (under 5) in the lower hierarchy. This governance indicator is followed by GE (49.519), with Singapore, Switzerland, Finland, Norway, Denmark as leaders (over 97), and Libya, Haiti, Somalia, Yemen, South Sudan at the bottom of the rankings (under 5). When it comes to the CC, the mean of all sample states is 48.160, with maximum values in Denmark, Finland, Singapore, New Zealand, Sweden (over 95) and minimum in Libya, Somalia, Yemen, Syria, South Sudan (under 5). Then, for the other three governance indicators, as a whole, the following averages are recorded: for the RL (48.102), V&A (47.581) and PS (44.715). Looking more closely at the differences between the groups of states, it can be seen that, at the level of high-income countries, on average, governance indicators have values above 80, except V&A (73.052) and PS (69.751); in the case of the middle-income ones, all governance indicators have values above 38, with GE reaching the highest level (43.055) and CC, the lowest (38.187); the low-income ones register the lowest values (the highest mean of 28.781 for V&A, and the lowest of 20.160 for GE). Considering the wide sample from our analysis, the scatterplots in Additional file 1: Appendix 1 show more clearly the positions of low, middle and high-income countries in terms of the relationship between COVID-19 vaccination and the six governance indicators. The ranking of the countries is calculated based on the scores recorded by the governance indicators on a scale from 0 to 100, where 100 means a very strong capacity of governments to ensure good governance. Shortly, it stands out that from the low-income countries group, those with the highest percentages of COVID-19 vaccination are: Rwanda (RWA), Bangladesh (BGD), Timor (TLS), Honduras (HND), and those with reduced vaccination rates are: Burundi (BDI), Democratic Republic of Congo (COD), South Sudan (SSD), Yemen (YEM), Haiti (HT). Among the countries included in the middle-income typology, the highest rates regarding COVID-19 vaccination are recorded by Cuba (CUB), China (CHN) and Cambodgia (KHM), and the lowest by Cameroon (CMR), Syria (SYR) and Djibouti (DJI). From the high-income group, United Arab Emirates (ARE), Brunei (BRN), Portugal (PRT), Chile (CHL) and Singapore (SGP) reach vaccination rates of over 80%. However, of the 53 countries included in this category, most have vaccination rates of over 60%. The states on the lower positions, Bahamas (BHS), Trinidad and Tobago (TTO), Slovakia (SVK), and Barbados (BRB) register percentages slightly below this margin. Analyzing the scatterplots, one can see the pronounced trend of having much higher rates of COVID-19 vaccination in high-income countries compared to low-income ones.

**Table 1:** Descriptive statistics (all sample, low, middle and high-income countries)

		COVID-19 vaccination (Dec.)	CC	GE	PS	RQ	RL	V&A	Comorb	GDP per capita	Stringency	COVID-19 deaths (Dec.)
All	Mean	49.257	48.160	49.519	44.715	49.745	48.102	47.581	18.688	19,398.45	57.149	728.613
	Max	98.990	100.000	100.000	97.641	100.000	100.000	100.000	42.700	116,935.6	87.960	6070.970
	Min	0.040	0.000	0.000	0.000	0.961	0.000	0.966	7.300	246.000	6.480	0.000
	Std. Dev	27.724	28.973	28.934	27.412	28.905	28.887	28.523	6.908	19,969.20	19.134	939.657
Low	Mean	16.540	24.433	20.160	22.023	22.467	22.329	28.781	24.017	2415.828	43.621	5.3052
	Max	57.330	69.711	64.423	53.301	58.173	57.211	60.869	42.700	6570.102	84.260	48.540
	Min	0.040	0.000	0.000	0.000	1.923	0.480	1.932367	16.100	246.000	6.480	0.000
	Std. Dev	15.770	17.471	14.340	16.465	13.554	14.473	15.557	5.727	1519.869	18.212	9.132
Middle	Mean	50.746	38.187	43.055	39.773	41.873	39.763	39.875	19.916	12,226.73	60.493	940.891
	Max	92.330	92.788	82.211	96.226	84.134	78.365	84.541	37.700	26,808.1	84.260	6070.97
	Min	2.960	0.480	1.923	0.471	0.961	0.000	0.966	9.500	2705.406	8.330	0.357
	Std. Dev	21.872	20.222	18.802	22.166	20.594	19.389	21.355	5.686	5596.017	17.874	1050.894
High	Mean	73.709	80.629	82.001	69.751	82.368	80.104	73.052	166.250	42,681.75	63.838	1042.520
	Max	98.990	100.000	100.000	97.641	100.000	100.000	100.000	350.060	116,935.6	87.960	4058.890
	Min	39.570	50.961	45.673	1.415	47.115	1.923	6.280	79.370	16,978.07	19.440	0.419
	Std. Dev	11.641	13.981	13.095	20.166	12.365	16.960	27.136	67.701	18,940.20	15.770	856.858

The indicator concerning the comorbidities is closely related to the COVID-19 vaccination rate: if the highest share of vaccination occurs in high-income countries, it seems that they also hold the greatest percentage of people with comorbidities, which explains, to some extent, the inter-conditionality between the two variables. Most probably, the percentage of people with comorbidities is higher in these states as a result of the influence of several factors, particularly related to: more frequent checking of the health status and registration in the national system of patients, pollution caused by urban agglomerations, the fast food diet, the fast pace of life and, implicitly, the daily stress. As vulnerable people, the vaccination process started with these population categories. Also, it is emphasized that the stricter the measures to fight against COVID-19 were (stringency index), the more the vaccination process accelerated, high-income countries registering the biggest values, followed by middle-income countries and low-income countries.

Then, by using OLS regression, the components of governance are analyzed separately, three at a time, for a better follow-up of the relationships they establish with COVID-19 vaccination. The estimates are presented in Tables 2 and 3. In connection with Table 2, this shows the regression results for the CC, GE and PS. For all models, the coefficients are positive and statistically significant (*p* < 0.05). The estimates for CC and GE are higher at the beginning of the period analyzed (in March 2021), so, even though,later, they slightly decrease, finally remain positive. The explanation for this may be that states that have more control over corruption turn out to have better health systems, transparent public policies, more responsible citizens, all these conducting to a higher vaccination rate. At the same time, the countries with lower corruption rates have more financial resources that can be directed to the medical system, for planning and recovery. Government effectiveness was the strongest in March (0.258), with a significance level of 0.009, this being justified by the fact that in the first months after the start of the vaccination campaigns and against the background of the disasters in 2020, there was an intensification of interest in COVID-19 vaccination. But, for this, the governments had to adapt and find the most effective ways to support the population in this process. It should be mentioned that the coefficients of this indicator are higher than those regarding the CC indicator in all four months.

**Table 2:** COVID-19 vaccination and CC, GE and PS (quarterly dynamics)

	Mar	Jun	Sept	Dec	Mar	Jun	Sept	Dec	Mar	Jun	Sept	Dec
COVID-19 vaccination (t-1)	0.364^c^ (.000)	0.846^c^ (.000)	0.947^c^ (.000)	0.960^c^ (.000)	0.377^c^ (.000)	0.831^c^ (.000)	0.955^c^ (.000)	0.958^c^ (.000)	0.378^c^ (.000)	0.821^c^ (.000)	0.946^c^ (.000)	0.961^c^ (.000)
CC	0.181^a^ (.014)	0.105^b^ (.005)	0.091^a^ (.029)	0.101^a^ (.044)								
GE					0.258^b^ (.009)	0.162^b^ (.009)	0.139^a^ (.050)	0.147^a^ (.023)				
PS									0.089^b^ (.002)	0.088^a^ (.027)	0.093^b^ (.003)	0.098^a^ (.013)
Comorb	0.362^a^ (.027)	0.048^b^ (.004)	0.094^a^ (.030)	0.016^b^ (.010)	0.402^a^ (.022)	0.039^b^ (.005)	0.090^a^ (.036)	0.018^b^ (.008)	0.388^a^ (.022)	0.048^a^ (.012)	0.109^a^ (.012)	0.024^a^ (.045)
GDP per capita	0.352^b^ (.007)	0.172^c^ (.000)	0.014^a^ (.045)	0.022^a^ (.011)	0.378^b^ (.006)	0.171^c^ (.000)	0.017^a^ (.037)	0.024 (.088)	0.318^a^ (.013)	0.163^c^ (.000)	0.011^a^ (.047)	0.023^a^ (.014)
COVID-19 deaths (t-1)	0.058^c^ (.001)	0.004^a^ (.046)	0.013^a^ (.013)	0.014^a^ (.050)	0.058^b^ (.009)	0.027^a^ (.050)	0.014^a^ (.011)	0.018^a^ (.041)	0.063^a^ (.025)	0.029^a^ (.041)	0.011^b^ (.003)	0.014^a^ (.049)
Stringency (t-1)	0.274^a^ (.028)	0.116^c^ (.062)	0.109^c^ (.002)	0.038^a^ (.014)	0.264^b^ (.008)	0.118^a^ (.042)	0.107^b^ (.003)	0.038^a^ (.014)	0.294^a^ (.024)	0.122^a^ (.050)	0.103^b^ (.005)	0.036^a^ (.016)
F-statistic	29.389	911.713	2154.650	2605.970	28.245	882.4232	2190.541	2619.518	30.867	886.212	2166.564	2677.132
Adjusted R-squared	0.512	0.971	0.987	0.989	0.502	0.970	0.987	0.989	0.523	0.970	0.987	0.989
Range of VIF value	1.033 2.765	1.489 3.918	1.712 3.829	1.596 3.438	1.156 2.764	1.489 3.770	1.712 4.170	1.594 3.448	1.140 2.537	1.486 3.869	1.716 4.030	1.599 3.370

^a^, ^b^, ^c^ indicates significance at the 10%, 5%, and 1% level

**Table 3:** COVID-19 vaccination and RQ, RL and V&A (quarterly dynamics)

	Mar	Jun	Sept	Dec	Mar	Jun	Sept	Dec	Mar	Jun	Sept	Dec
COVID-19 vaccination (t-1)	0.372^c^ (.000)	0.832^c^ (.000)	0.951^c^ (.000)	0.963^c^ (.000)	0.365^c^ (.000)	0.837^c^ (.000)	0.947^c^ (.000)	0.962^c^ (.000)	0.390^c^ (.000)	0.830^c^ (.000)	0.947^c^ (.000)	0.965^c^ (.000)
RQ	0.296^a^ (.044)	0.089^a^ (.011)	0.161^a^ (.019)	0.158^a^ (.028)								
RL					0.145^a^ (.011)	0.086^a^ (.022)	0.066^b^ (.008)	0.082^a^ (.013)				
V&A									0.123^a^ (.020)	0.104^c^ (.001)	0.075^a^ (.040)	0.071^a^ (.036)
Comorb	0.429^a^ (.019)	0.058^a^ (.042)	0.103^a^ (.017)	0.023^a^ (.047)	0.425^a^ (.020)	0.056^a^ (.038)	0.109^a^ (.012)	0.025^a^ (.039)	0.254^a^ (.045)	0.113^a^ (.013)	0.097^a^ (.032)	0.029^a^ (.038)
GDP per capita	0.367^b^ (.005)	0.172^c^ (.000)	0.016^a^ (.039)	0.023^b^ (.010)	0.388^b^ (.004)	0.167^c^ (.000)	0.019^a^ (.050)	0.026^a^ (.047)	0.461^c^ (.000)	0.143^c^ (.000)	0.011^a^ (.050)	0.018^a^ (.017)
COVID-19 deaths (t-1)	0.043^a^ (.044)	0.026^b^ (.005)	0.011^a^ (.023)	0.014^a^ (.043)	0.056^a^ (.032)	0.049^a^ (.030)	0.018^a^ (.024)	0.013^a^ (.048)	0.018^a^ (.044)	0.084^a^ (.055)	0.099^a^ (.030)	0.016^a^ (.037)
Stringency (t-1)	0.333^a^ (.019)	0.103^a^ (.015)	0.096^a^ (.010)	0.036^a^ (.014)	0.268^a^ (.036)	0.122^a^ (.050)	0.098^b^ (.008)	0.031^a^ (.025)	0.311^a^ (.022)	0.114^a^ (.047)	0.102^b^ (.005)	0.034^a^ (.019)
F-statistic	28.910	914.375	2189.990	2659.469	28.001	928.237	2187.620	2658.343	29.373	937.550	2175.863	2654.014
Adjusted R-squared	0.506	0.970	0.987	0.989	0.515	0.971	0.987	0.989	0.510	0.971	0.987	0.989
Range of VIF value	1.163 2.631	1.512 3.750	1.743 3.831	1.617 3.494	1.180 2.726	1.507 3.881	1.707 3.802	1.588 3.617	1.140 2.158	1.488 3.503	1.442 3.548	1.497 3.268

^a^, ^b^, ^c^ indicates significance at the 10%, 5%, and 1% level

As for PS, this indicator has the lowest values in relation to COVID-19 compared to all governance variables, which means that, on the one hand, in our sample, there could be fewer states animated by internal violence and political instability and that, on the other hand, even so, they would not greatly influence the intention to vaccinate if access to vaccines is provided.

In Table 3, the results for the other three governance indicators, namely RQ, RL and V&A are presented. Analogous, the coefficients are on the same trend, meaning positive and statistically significant (*p* < 0.05), with the highest values in March, then, they decrease marginally.

The variable pertaining to RQ has the greatest interconditionality with COVID-19 vaccination rate. The government’s ability to formulate clear and unambiguous policies and regulations are particularly relevant [65]. During the COVID-19 crisis, governments were practically forced to propose and adopt extremely cumbersome legislation overnight in most of the citizens’ activities [66]. Under these conditions, high regulatory quality is essential for the proper and timely implementation of public health policies. It is also notable that for these three governance indicators in Table 3, the highest coefficients were recorded in March, which highlights, once again, the fact that without adequate regulations, compliance with laws and the responsible involvement of institutions and citizens, the process of COVID-19 vaccination would have been affected since its start, which would have been undesirable especially in the context where, in 2021, the dominant variant was Delta, known as the most severe form of this disease, causing the most deaths, according to the WHO.

### Panel regressions

The panel regression is used to emphasize the influence of governance indicators at the level of our sample, namely 170 countries, previously determined with the help of the OLS model. A clearer differentiation regarding the established relationships between COVID-19 vaccination and governance indicators in low, middle and high-income countries can be observed in Tables 4 and 5.

**Table 4:** COVID-19 vaccination and governance (CC, GE and PS) for low, middle and high-income countries

	Low	Middle	High	All	Low	Middle	High	All	Low	Middle	High	All
COVID-19 vaccination (-1)	0.363^c^ (.000)	0.732^c^ (.000)	0.603^c^ (.000)	0.713^c^ (.000)	0.359^c^ (.000)	0.728^c^ (.000)	0.603^c^ (.000)	0.721^c^ (.000)	0.358^c^ (.000)	0.721^c^ (.000)	0.604^c^ (.000)	0.718^c^ (.000)
CC	0.115^b^ (.001)	0.132^a^ (.023)	0.228^a^ (.021)	0.122^b^ (.005)								
GE					0.059^b^ (.009)	0.120^b^ (.002)	0.198^a^ (.024)	0.148^a^ (.034)				
PS									0.093^a^ (.021)	0.088^b^ (.002)	0.107^a^ (.016)	0.093^a^ (.041)
Comorb	1.421^c^ (.000)	0.261^a^ (.011)	0.191^a^ (.023)	0.111^a^ (.038)	1.311^c^ (.000)	0.271^a^ (.029)	0.251^a^ (.014)	0.127^a^ (.018)	0.402^c^ (.000)	0.227^a^ (.016)	0.299^a^ (.016)	0.118^a^ (.035)
GDP per capita	0.185^a^ (.014)	0.159^a^ (.013)	0.338^c^ (.010)	0.265^c^ (.000)	0.225^a^ (.047)	0.157^a^ (.013)	0.291^a^ (.030)	0.216^c^ (.000)	0.158^a^ (.019)	0.199^a^ (.050)	0.266^a^ (.045)	0.240^c^ (.000)
Stringency (t-1)	0.165^c^ (.000)	0.119^a^ (.029)	0.279^a^ (.016)	0.005^c^ (.000)	1.184^c^ (.000)	0.130^a^ (.025)	0.235^a^ (.024)	0.005^c^ (.000)	1.192^c^ (.000)	0.148^a^ (.018)	0.233^a^ (.025)	0.005^c^ (.000)
COVID-19 deaths (t-1)	0.016^a^ (.050)	0.016^a^ (.035)	0.072^a^ (.011)	0.204^c^ (.000)	0.014^b^ (.006)	0.017^a^ (.037)	0.072^a^ (.011)	0.205^c^ (.000)	0.018^a^ (.042)	0.022^a^ (.042)	0.071^a^ (.012)	0.205^c^ (.000)
Adjusted R-squared	0.497	0.759	0.711	0.585	0.501	0.761	0.704	0.585	0.497	0.766	0.704	0.584
Countries	45	72	53	170	45	72	53	170	45	72	53	170
N	180	288	212	680	180	288	212	680	180	288	212	680
Country FE	yes	yes	yes	yes	yes	yes	yes	yes	yes	yes	yes	yes

^a^, ^b^, ^c^ indicates significance at the 10%, 5%, and 1% level

**Table 5:** COVID-19 vaccination and governance (RQ, RL and V&A) for low, middle and high-income countries

	Low	Middle	High	All	Low	Middle	High	All	Low	Middle	High	All
COVID-19 vaccination (-1)	0.354^c^ (.000)	0.734^c^ (.000)	0.601^c^ (.000)	0.709^c^ (.000)	0.347^c^ (.000)	0.733^c^ (.0 00)	0.604^c^ (.000)	0.718^c^ (.000)	0.354^c^ (.000)	0.737^c^ (.000)	0.596^c^ (.000)	0.716^c^ (.000)
RQ	0.086^a^ (.046)	0.172^a^ (.021)	0.290^a^ (.027)	0.162^a^ (.014)								
RL					0.061^a^ (.041)	0.073^a^ (.026)	0.105^a^ (.050)	0.081^a^ (.042)				
V&A									0.025^b^ (.001)	0.094^a^ (.012)	0.104^a^ (.026)	0.073^c^ (.000)
Comorb	1.446^c^ (.000)	0.264^a^ (.019)	0.198^a^ (.027)	0.113^a^ (.036)	1.282^a^ (.000)	0.250^a^ (.012)	0.284^b^ (.006)	0.138^a^ (.027)	0.505^c^ (.000)	0.212^a^ (.019)	0.481^a^ (.012)	0.021^b^ (.006)
GDP per capita	0.169^a^ (.022)	0.158^a^ (.013)	0.308^a^ (.022)	0.297^c^ (.000)	0.215^a^ (.019)	0.164^a^ (.012)	0.290^a^ (.032)	0.233^c^ (.000)	0.100^a^ (.042)	0.178^a^ (.039)	0.339^a^ (.012)	0.248^c^ (.000)
Stringency (t-1)	0.178^c^ (.000)	0.148^a^ (.019)	0.261^a^ (.019)	0.582^c^ (.000)	1.180^c^ (.000)	0.125^a^ (.027)	0.208^a^ (.031)	0.565^c^ (.000)	1.191^c^ (.000)	0.128^a^ (.025)	0.232^a^ (.024)	0.560^c^ (.000)
COVID-19 deaths (t-1)	0.017^a^ (.050)	0.012^a^ (.016)	0.069^a^ (.013)	0.207^c^ (.000)	0.006^a^ (.044)	0.017^a^ (.033)	0.073^a^ (.011)	0.206^c^ (.000)	0.021^a^ (.022)	0.004^a^ (.017)	0.089^a^ (.050)	0.216^c^ (.000)
Adjusted R-squared	0.494	0.760	0.705	0.593	0.502	0.759	0.704	0.589	0.492	0.760	0.708	0.596
Countries	45	72	53	170	45	72	53	170	45	72	53	170
N	180	288	212	680	180	288	212	680	180	288	212	680
Country FE	yes	yes	yes	yes	yes	yes	yes	yes	yes	yes	yes	yes

^a^, ^b^, ^c^ indicates significance at the 10%, 5%, and 1% level

For CC, the coefficient for the entire sample is positive (0.122) and statistically significant (*p* < 0.05); the same is obtained in the case of high-income countries (0.228), middle-income (0.132) and low-income (0.115) countries. For the GE indicator, the estimates are also positive: 0.148 for all countries; 0.198 for high-income countries; 0.120 for middle-income and 0.059 for low-income. Practically, the two indicators (CC and GE) are strongly linked, because corruption can be reduced only under the conditions of the existence of sound institutions. Regarding PS, a quite similar trend is found: high-income countries are more stable from this point of view, just as the highest levels of corruption control and government efficiency meet here. However, among the three governance indicators presented in Table 4, PS has the least influence on the COVID-19 vaccination.

In Table 5, the connection of the other three governance indicators (RQ, RL and V&A) with the COVID-19 vaccination is pointed out.

The coefficients for RQ are positive: for all panel (0.162), high-income countries (0.290), middle-income (0.172) and low-income (0.086). In this case, the values obtained from the analysis show the particular usefulness of the rapid adoption and implementation of a legislative framework conducive to combating the pandemic. The regulatory setting matters both in terms of reducing cases and deaths caused by COVID-19, but also more importantly in terms of the opportunity to take appropriate measures. The speed of the nations’ responses also depended on the government’s ability to quickly adopt regulations and to put them into practice, in order to coordinate the new realities. The prompt reaction of regulatory authorities by issuing relevant guidelines during the severe phase of the pandemic could have ensured that quality requirements were met throughout it, to save as many lives as possible, but, unfortunately, for most low-income countries, the legislative aspects, respecting the rule of law and those regarding responsible actions had many gaps, a fact confirmed by the coefficients presented in Table 5. High-income countries have the most significant values of the governance indicators, this proving a high capacity of these states to support the vaccination process. However, considering that of the 170 countries studied, most (72 countries) are included in the middle-income typology, as a whole (cumulative for the quarters of 2021), the estimates of COVID-vaccination are the highest for this category of states (coefficient over 0.720).

More specifically, certain particularities can be distinguished in the three groups of countries included in our analysis:

- ▪ for *low-income countries*, the order of influence of governance indicators on COVID-19 vaccination is: CC (0.115), PS (0.093), RQ (0.086), RL (0.061), GE (0.059) and V&A(0.025);
- ▪ in the case of *middle-income countries*, the following hierarchy of governance indicators in the COVID-19 vaccination process is emphasized: RQ (0.172), CC (0.132), GE (0.120), V&A (0.094), PS (0.088) and RL (0.073).
- ▪ in the COVID-19 vaccination process, in *high-income countries*, it stands out as relevant: RQ (0.290), CC (0.228), GE (0.198), PS (0.107), RL (0.105) and V&A (0.104).

Therefore, even if all governance indicators have proven to have a direct influence on COVID-19 vaccination, each country category should adapt the institutional arrangements in order to strengthen its weaker components, which would mean real advantages in public health terms. According to the results, it is observed that more attention should be paid to the involvement and responsibility of the population in the face of crises (voice & accountability) and to promoting the importance of respecting the rule of law everywhere.

### Robustness tests

The robustness of the results obtained by OLS regressions and panel model was also tested. The methodology used is the same with the previous one, but changing one variable with another one: the variable related to comorbidities was replaced by that of the population over 65 years old. We can talk about suitable initial models if it turns out that this indicator will have an explicit influence on vaccination cases. We chose this one because, in most countries, the vaccination process started with this population category, which is more exposed to the negative consequences of COVID-19. In Tables 6 and 7, the robustness between COVID-19 vaccination and the six governance indicators are presented. In the second stage of robustness testing, we use the panel model. The results are broadly in line with those gathered in the first part of the analysis; they are positive and significant.

**Table 6:** Robustness check of the model for COVID-19 vaccination and governance (CC, GE and PS)

	Low	Middle	High	All	Low	Middle	High	All	Low	Middle	High	All
COVID-19 vaccination (-1)	0.345^c^ (.000)	0.733^c^ (.000)	0.602^c^ (.000)	0.715^c^ (.000)	0.343^c^ (.000)	0.729^c^ (.000)	0.596^c^ (.000)	0.724^c^ (.000)	0.340^c^ (.000)	0.721^c^ (.000)	0.613^c^ (.000)	0.720^c^ (.000)
CC	0.121^a^ (.018)	0.106^a^ (.031)	0.193^c^ (.000)	0.119^a^ (.036)								
GE					0.064^b^ (.005)	0.114^a^ (.014)	0.155^c^ (.000)	0.161^a^ (.027)				
PS									0.103^b^ (.007)	0.072^b^ (.002)	0.101^a^ (.044)	0.010^a^ (.012)
Population 65 +	0.203^c^ (.000)	0.116^a^ (.012)	0.373^c^ (.000)	0.119^a^ (.010)	0.084^b^ (.002)	0.279^a^ (.012)	0.392^c^ (.000)	0.143^a^ (.048)	0.230^c^ (.000)	0.112^a^ (.020)	0.143^a^ (.051)	0.133^a^ (.046)
GDP per capita	0.080 (.055)	0.167^a^ (.014)	0.677^c^ (.000)	0.265^c^ (.000)	0.137^a^ (.032)	0.175^a^ (.012)	0.706^c^ (.000)	0.218^c^ (.000)	0.067^a^ (.050)	0.216^a^ (.038)	0.333^a^ (.020)	0.242^c^ (.000)
Stringency (t-1)	0.154^c^ (.000)	0.143^a^ (.021)	0.538^b^ (.003)	0.566^c^ (.000)	0.980^c^ (.000)	0.150^b^ (.018)	0.410^a^ (.026)	0.572^c^ (.000)	0.975^c^ (.000)	0.165^a^ (.014)	0.418^a^ (.029)	0.569^c^ (.000)
COVID-19 deaths (t-1)	0.020^a^ (.027)	0.020^a^ (.048)	0.064^a^ (.011)	0.217^c^ (.000)	0.018^a^ (.048)	0.022^a^ (.028)	0.077^b^ (.007)	0.220^c^ (.000)	0.021^a^ (.022)	0.026^a^ (.034)	0.043^a^ (.036)	0.219^c^ (.000)
Adjusted R-squared	0.484	0.757	0.735	0.591	0.490	0.759	0.724	0.591	0.488	0.764	0.704	0.591
Countries	45	72	53	170	45	72	53	170	45	72	53	170
N	180	288	212	680	180	288	212	680	180	288	212	680
Country FE	yes	yes	yes	yes	yes	yes	yes	yes	yes	yes	yes	yes

^a^, ^b^, ^c^ indicates significance at the 10%, 5%, and 1% level

**Table 7:** Robustness check of the model for COVID-19 vaccination and governance (RQ, RL and V&A)

	Low	Middle	High	All	Low	Middle	High	All	Low	Middle	High	All
COVID-19 vaccination (-1)	0.335^c^ (.000)	0.749^c^ (.000)	0.584^c^ (.000)	0.712^c^ (.000)	0.328^c^ (.001)	0.769^c^ (.000)	0.611^c^ (.000)	0.721^c^ (.000)	0.334^c^ (.000)	0.758^c^ (.000)	0.616^c^ (.000)	0.717^c^ (.000)
RQ	0.071^a^ (.049)	0.161^a^ (.029)	0.233^c^ (.000)	0.091^a^ (.027)								
RL					0.094^a^ (.022)	0.058^a^ (.032)	0.106^b^ (.010)	0.092^a^ (.049)				
V&A									0.014^b^ (.004)	0.102^b^ (.002)	0.119^a^ (.035)	0.050^b^ (.001)
Population 65 +	0.227^c^ (.000)	0.117^a^ (.029)	0.378^c^ (.000)	0.099^a^ (.017)	0.094^b^ (.002)	0.115^a^ (.031)	0.168^a^ (.022)	0.136^b^ (.006)	0.284^c^ (.000)	0.121^b^ (.011)	0.326^b^ (.006)	0.051^c^ (.005)
GDP per capita	0.049^a^ (.037)	0.168^a^ (.014)	0.278^c^ (.000)	0.164^c^ (.000)	0.138^a^ (.030)	0.170^a^ (.013)	0.398^b^ (.007)	0.236^c^ (.000)	0.095^a^ (.013)	0.164^a^ (.011)	0.358^b^ (.008)	0.259^c^ (.000)
Stringency (t-1)	0.154^c^ (.000)	0.168^a^ (.014)	0.270^b^ (.010)	0.185^c^ (.000)	0.980^c^ (.000)	0.147^a^ (.019)	0.252^b^ (.007)	0.572^c^ (.000)	0.952^c^ (.000)	0.147^a^ (.019)	0.365^a^ (.050)	0.559^c^ (.000)
COVID-19 deaths (t-1)	0.022^a^ (.014)	0.017^a^ (.054)	0.071^b^ (.008)	0.217^c^ (.000)	0.007^a^ (.041)	0.020^a^ (.047)	0.047^a^ (.027)	0.220^c^ (.000)	0.025^b^ (.009)	0.005^a^ (.030)	0.038^a^ (.042)	0.220^c^ (.000)
Adjusted R-squared	0.480	0.757	0.727	0.593	0.495	0.757	0.707	0.591	0.479	0.759	0.709	0.596
Countries	45	72	53	170	45	72	53	170	45	72	53	170
N	180	288	212	680	180	288	212	680	180	288	212	680
Country FE	yes	yes	yes	yes	yes	yes	yes	yes	yes	yes	yes	yes

^a^, ^b^, ^c^ indicates significance at the 10%, 5%, and 1% level

The previously obtained observations are generally kept: high-income countries show higher coefficients for all governance indicators (except for PS). In the case of the low-income countries, sometimes, the results, although positive, are not so significant. For CC, for all countries (0.119), high-income (0.193), middle (0.106), low (0.121), the coefficients are positive. For GE, the results obtained (all countries 0.161, high 0.155, middle 0.114, low 0.064) are similar to those prior achieved. Even if in terms of PS, the differences are not very wide between high and low-income countries (0.101 versus 0.103), we can explain this by the fact that, in some developed countries, against the background of quite severe restrictions, which culminated with the total lockdown, discontent arose among the population and the business environment, which also resulted in mass protests and internal violence.

Looking at the other three governance indicators in Table 7, it is established that high-income countries have the greatest values: for RQ (0.233), RL (0.106) and V&A (0.119), the highest significance level being in the case of RQ.

If we refer to the entire sample, the robustness coefficients are as follows (in ascending order): for PS (0.010), V&A (0.050) RQ (0.091), RL (0.092), CC (0.119) and GE (0.161). The lowest values are associated with PS, respectively V&A, probably in the context in which the citizens fully felt the government measures imposed, some of them very restrictive, which led to widespread dissatisfaction, primarily related to freedom of action. For Western societies and not only, this may mean a violation of basic liberties, a fact for which there have been disturbances, political as well, in the systems.

## Discussion

Good governance, perceived as the set of processes and tools through which steps towards recovery and development can be generated, should be accompanied by responsibility, transparency, uprightness and leadership. All of this falls, in particular, on the institutions and decision-making actors. Therefore, the identification of vulnerabilities and the analysis of governments’ ability to reduce the harmful effects of crises should be priorities on a large scale. In general, health systems around the world are affected differently by governance, this modeling the long-term development outcomes in this sector.

The findings highlighted that the governance indicators influence the COVID-19 vaccination process, the most significant for the analyzed period (quarters of 2021) being, in order of their importance: GE, RQ, CC, RL, V&A and PS. Thus, in the current context, the quality of public health policies is primarly conditioned by the government effectiveness, which substantially transpose in the validity of the responses offered to the COVID-19 crisis. Government effectiveness remains critical to responding to the COVID-19 pandemic and immunizing the population (the scores obtained have a strong and positive impact on vaccination rates). The efficiency of the government is paramount in taking other measures with major influence on the evolution of the pandemic, such as the coordinated logistical planning for vaccination, which can include COVID-19 testing, ongoing monitoring of personal protective equipment, adequate medical supplies, good organization for the distribution of vaccines, even in difficult geographical areas (e.g., those affected by war or instability), effective management and treatment of patients with COVID-19.

Although different factors may contribute to counteracting the diffusion of COVID-19, good governance has a special role to play because it means implementing those public strategies and policies that minimize the negative repercussions of the pandemic [67]. Institutions can shape how things can be done in society. For this reason, if there is effective cooperation and coordination between the stakeholders involved (governments, local authorities, public health directorates, private sector, medical research entities, hospitals, international institutions), the public’s confidence in accepting and respecting the rule of law will be higher. As noted, there is a tendency for higher levels of acceptance of vaccination where governance also has greater values, and this is precisely due to trust in institutions and the existence of social dialogue based on truthful and complete information, to make citizens aware of both the benefits and possible side effects of vaccines. During crises, the good governance is more relevant than ever, and, in the COVID-19 context, it mainly means to inform the population about the possible risks that may arise: overcrowding of hospitals, increasing the likelihood of potentially more severe mutations that do not respond to existing vaccines, pressure on medical staff, depletion of resources to support health systems [35]. The governments should have the capacity to support the efforts and achievements of scientists, to communicate effectively with citizens to ensure vaccination campaigns with optimal results, thus eliminating misinformation. While young people are more resistant to this virus, especially if comorbidities are ruled out, for older people, who often have certain medical conditions, it is more than necessary to become acquianted with the benefits of immunization by vaccination.

However, the results of the COVID-19 vaccination process were different for each category of countries analyzed (high, middle and low-income). In low-income countries, where access to immunization facilities is quite difficult due to many marginalized groups, the adequate distribution of vaccines has been a challenge. Also, for these category of countries, in particular, the lack or precariousness of the necessary means for the transport and storage of vaccines should be mentioned, considering that they should be kept at very low temperatures. In certain low and middle-income countries (India, Brazil, Indonesia, South Africa), the production of COVID-19 vaccines was carried out [68], but many of them do not have the internal capacity to manufacture vaccines and thus rely on high-income countries or multinational companies. Moreover, they show pronounced income inequalities, deficiencies in resources, government effectiveness and regulatory quality such as policies, planning, programmes, medical personnel, organized laboratories, industries, research & development, and government funding. Even if these countries were granted adequate funding and access to vaccine patents, in the absence of strong government effectiveness, the problems of vaccine production and timely immunization of the population could not be properly resolved, countries thus facing significant delays in vaccine access, despite initiatives aimed at ensuring equitable distribution, such as COVID-19 Vaccines Global Access (COVAX) [69]. In the case of low and middle-income countries, the WHO tried to establish a COVAX intra-aid tool, to access doses of vaccines, external financing being necessary for purchases or donations [52, 70]. An issue of major differentiation in regulatory quality regarding COVID-19 vaccines was the authorization of vaccines: while the high-income countries quickly authorized them, the low and middle ones did not emit the necessary licenses for their delivery and widespread use in a timely manner [71]. Another governance problem arising in the population vaccination process in some low and middle-income countries is the poor inclusion of people most exposed to the COVID-19 (population with comorbidities and those aged over 65) in the vaccination campaigns. For this reason, governments should demonstrate transparency and good regulatory quality when they take measures and should, furthermore, rely on scientific evidence when explaining the priority vaccination of certain population categories and thus build confidence in COVID-19 vaccines [68, 72].

If we were to compare the three groups of countries studied, it turns out that for those in the low-income category, the control of corruption matters the most in the COVID-19 vaccination process, and for both middle and high-income countries, the most relevant governance indicator is represented by the regulatory quality. These findings can be explained by the fact that, on the one hand, countries with lower incomes cannot afford to generalize effective mechanisms for identifying and sanctioning corruption, which slows down the vaccination process, and, on the other hand, middle- and high-income countries make efforts to adapt their legislation to fluctuating contexts, emphasizing the need for the existence of quality regulations so that they reach the best results. Knowing the European specifics better and judging by the experience gained over the years, the citizens of countries that have gone through an oppressive regime (in the East) may prove, in some cases, to no longer readily accept directives to change behaviour. But, at the same time, being already used to restrictions, they can master them faster compared to the population in western societies. It should also be emphasized that the governance indicator with the lowest values in association with the COVID-19 vaccination process is voice & accountability, both in the case of low and of high-income countries, which shows that citizens, in general, could not be involved too much in the decision-making process, as it is an issue of public health that should rather be adapted to international norms and directives.

Debates surrounding the COVID-19 vaccination have included, quite often, issues related to distribution, equity and respect for social rights [73]. However, there are significant gaps between states in terms of these aspects, which reinforces the need for organizations or governments to act on rationales connected to solidarity, especially when it comes to a global public health problem, which should not differentiate between people. Even if there is a pronounced distance regarding the COVID-19 vaccination between high-income countries of the world and those with internal deficiencies, international institutions should support the latter so that everyone could benefit from the fundamental right to have access to health services. In order to know where an urgent intervention is required, states should have effective reporting systems that allow the recording of statistical data on a regular basis. There are isolated communities and countries without close international ties, and in the absence of homogeneous data, it is very difficult to quantify the plurality of facets of the COVID-19 vaccination process (for instance, the distribution of COVID-19 vaccines by age groups, gender, population category, medical conditions, prevention behaviors, race, or place of origin). Therefore, a more extensive analysis, starting from these criteria, could not be realized, because there are no such integrated data worldwide, at least as far as we know, to cover the chosen sample (170 states). There are, however, certain statistics addressing the specifics of some communities, countries or regional groups, related to COVID-19: e.g., the US Department of Health and Human Services, through the Centers for Disease Control and Prevention (CDC), provides the population and the authorities with weekly updated statistics in relation to COVID-19 vaccination equity (for racial and ethnic minority groups), but only for the US [74].

The lack of a reliable ongoing data collection system, based on reports guided by the same criteria, causes discontinuities in the time series and makes it intricate to cover wider territorial issues in connection with COVID-19 vaccination [75]. To have an aggregate reporting system, several measurement criteria should have been set at the international level and applied for all the countries of the world. Moreover, in order to act promptly on the basis of the alert systems and to account for the progress in terms of vaccination, these reports should be made at least weekly, divided mainly by categories of groups, age, race and ethnicity, as it is suggested by ‘Prevent Epidemics’ [76]. Without such statistics and without considering a unitary and transparent evaluation system, it is impossible to precisely quantify (in)equity in the distribution of COVID-19 vaccines.

The obstacles that intervene in the collection of uniform data by country, which would allow comparisons to be made, are mainly related to the legislative aspects, to the digital infrastructure of the states, to what is understood by some indicators (for example, [76] signaled that “doses received” means, on the one hand, the COVID-19 doses distributed to the US jurisdictions, and, on the other hand, the COVID-19 doses administered (partially vs. fully), which leads to inconsistent reporting). Moreover, the United States Agency for International Development (USAID) supported the initiative regarding global access to COVID-19 vaccines, offered technical assistance for their delivery and proposed a compendium with a series of indicators related to vaccination (e.g., the number of guides, plans and strategies; number of people trained; number of countries with vaccine tracking systems; the number of people reached by USAID messages regarding COVID-19 vaccinations through various information channels, etc.), but all of them are addressed to the US [77].

Therefore, guiding the distribution of COVID-19 vaccines based on public statistical data should be of particular interest for governments, all the more so as there were countries that purchased a much larger number of vaccine doses (Australia, Canada, Great Britain) and later, they had to redistribute or donate them to other countries in need [78]; there were also situations where vials were wasted for various reasons [79, 80]. Several studies clearly emphasize that the access to COVID-19 vaccines is facilitated by favorable socio-economic conditions [81], and the priority target group in obtaining them should be the population at major risks [82, 83]. Thus, low-income countries should be generously supported to reach a significant immunization threshold and to have an equitable vaccination so that marginalized groups are not extremely affected [84, 85]. Although the coverage of COVID-19 vaccination is quite heterogeneous globally, equitable access to vaccines for low- and middle-income states has been shown to be shaped by global mechanisms, prices, know-how, and the ability to carry out strong vaccination programs [86]. In addition, in order to reduce the disproportionate access to vaccines, [87] advocate for a Universal Vaccine Access Strategy, through which shortages in vulnerable countries can be adjusted, including by donating unused vials in countries that have purchased vaccines in excess of their needs. Then, the WHO, the United Nations Children’s Fund (UNICEF) and the Vaccine Alliance (GAVI) set up the COVID-19 Vaccine Delivery Partnership (CoVDP), in January 2022, to support low-income countries in the vaccination process, and through this initiative, 34 countries, especially in Africa, which recorded, in January 2022, vaccination rates lower than 10%, were helped to have access to the COVID-19 vaccine [88]. Through this laudable initiative, vaccination noticeably improved: e.g., Ethiopia reached a vaccination rate of over 33% in July 2022, while in January 2022 it had a rate of only 3%; then, more than 8 countries out of the 34 backed up by CoVDP exceeded 20% COVID-19 vaccination rates [89]. Then, we should also mention UNICEF’s efforts to establish the Access to Covid Tools Accelerator Supplies Financing Facility (ACT-A SFF) to help the poorest countries in the procurement of vaccines [90, 91]. Therefore, if in the case of high-income countries, which have more solid health systems, more technologically advanced medical infrastructure, COVID-19 tracking tools, it is easier to collect statistical data, in the case of low-income countries, given the problems they faced, it is demanding for them to have a broad coverage regarding the delivery of COVID-19 vaccines and precisely for this reason international support is crucial [92]. Even if in the year 2022 a slight improvement was made regarding COVID-19 vaccination, compared to the year 2021, within low-income groups (Additional file 1: Appendix 2) [93], actions to reduce imbalances between states should continue, primarily pursuing the right to health of each individual.

Anyway, the application of extensive studies that highlight the various drivers of COVID-19 vaccination could better shape immunization policies in low-income countries; e.g., [94] conducted a research on several countries, including some in this category, such as Burkina Faso, Mozambique, Rwanda, Sierra Leone, Uganda, and they found that in Burkina Faso there is the lowest acceptance rate when it comes to the COVID-19 vaccine (66.5%); therefore, it is necessary to take specific measures in the direction of identified weaknesses.

In our analysis, only a small part of the broad palette of elements that can explain the dynamics of the COVID-19 vaccination process were captured. Therefore, there are certain limits of the research related to the fact that other indicators could have been taken into account, such as: the endowment of countries with special equipment for transporting and storing vaccines, the number of vaccination campaigns, international financial support, the level of education, all these being crucial, especially in the case of low and middle-income countries. Moreover, considering that the variables that define good governance (WGI of the World Bank) are expressed in perception data, it would have been interesting to apply opinion polls both among the population and health risk experts, to homogenize measurement units and to prevent possible biases between indicators. However, it would be almost impossible to cover a very large sample through surveys, which would distinguish the particularities of each country. This can be achieved, instead, in extended research teams and with governmental support.

## Conclusions

The COVID-19 crisis intensified the concerns related to how this is reflected on different dimensions and geographical spaces. The actionable tools are shaped, most of the times, by the available resources, but also by the capacity of institutions to address suitable measures to monitor the progress made in terms of recovery and to raise awareness regarding the severity of a pandemic. Since the outbreak of this health crisis, we have tried, in various works, to deal with this topic dynamically, in order to emphasize the way in which COVID-19 spreads over the nations as a whole, as well as its intricate impact [95, 96]. This time, our focus was on the relationship between COVID-19 vaccination and governance. Thus, advocating for the relevance of governance structures in any major crisis, this paper pointed out that the defining elements of governance imprint the COVID-19 immunization process. As a result of both the numerous cases and deaths of COVID-19 reported globally in 2020, WHO, national governments, along with other regional and local institutions have joined their efforts to fight against the COVID-19 pandemic. The best way to control and stop this disease is to gain mass immunity, especially with safe and effective vaccines. Thus, the governments of the world’s states and scientists have concentrated their attention to develop a vaccine against COVID-19 and, one year after the onset of this health crisis, several vaccines have been proposed.

Public discourse should focus on the scientific component, in the sense that medical research has undergone remarkable developments in recent decades, without which humanity would have been faced with many life-threatening dangers. In addition, technological and informational progress can shorten the period of introduction of drugs or injectable solutions on the market. Therefore, there should be significant support for health from all governments, by allocating expenditure that can cover not only basic public services but also advanced medical research. As long as the discovery of vaccines means offering the possibility to have the right to life for a considerable number of people, especially the vulnerable ones, this is worth massive investments. Therefore, support efforts from state institutions should be constant in this direction. All vaccines against COVID-19 have been based on scientific evidence. There have been countries that have overwhelmingly accepted immunization by vaccination, and there have also been states that have been more open to natural immunization. In the context where it is demonstrated that the Omicron variant is more contagious, but less virulent, as compared to previous strains (Alpha, Delta, etc.), there is a tendency for vaccination campaigns to be affected. For some people, who have not been vaccinated until finding a reduction in the severity of cases associated with Omicron, the probability of postponing vaccination precisely for this reason could be high.

By referring to the main results of our study, it turns out that, at the level of the 170 countries analyzed, the interaction between the vaccination rate and the governance dimensions was positive in 2021, their order of influence on COVID-19 immunization being: GE, RQ, CC, RL, V&A and PS. Therefore, even if, for example, the quality of regulations in a country is high, leading to a good quality of public services and policies, one can also discuss about the control of corruption, which strengthens the population’s trust in government and other state institutions, or about the usefulness of respecting the rule of law, all together shaping the degree of vaccination. The behaviour of individuals is not expected to be modeled to a large extent by the political component. Overall, countries with better governance are associated with higher levels of COVID-19 vaccination, compared to those with weaker institutions. In the case of low-income countries, there are certain blockages in the medical system due to financial reasons. The special handling and transport requirements for COVID-19 vaccines, which translates in the use of low temperatures, make it extremely difficult to vaccinate the population in these countries. Considering previous experiences, the immunization infrastructure is broadly inadequate and vaccinating the population for COVID-19 requires considerable investments of material and human resources, as well as careful planning to avoid potential problems (e.g., interruption of routine health services). Moreover, interests groups and corruption severely undermine public actions, or, in the case of the COVID-19 pandemic, prompt and very rapid responses to the changes caused by the disease are needed [97]. Given these, the governments from low-income countries should stay focused on increasing the efficiency of public health policies aimed at the COVID-19 vaccination process and on reducing inequities in terms of access to the vaccine. There is a significant gap between these and high-income countries in terms of COVID-19 vaccination but, beyond drawing policies that objectively reflect the degree to which they have resources and action tools to fight against shocks, compelling efforts are needed at all levels. On this line, the ability of governments to respond to the challenges in accordance with the needs of the citizens, to ensure correct and transparent information, to increase the confidence in the actions to be put into practice, as well as to pursue coordinated strategies to obtain satisfactory results prevails, as many studies have shown [98–102]. Governments and their leaders should not show excessive authority, which creates the impression of annulment of individual freedom, but rather to provide the premises of a partnership with individuals and other decision-makers. In addition, the responsibility should be shared among all those involved. Definitely, medical institutions, which coordinate the combined efforts and give the best direction to follow should be the centre of these measures. Thus, people are more likely to accept the messages of those involved in vaccination campaigns; also, some solutions can be outlined and, even though they may not be immediately implemented, they may, at least provide a certain degree of predictability. Large-scale vaccination may place us near the total end of the COVID-19 pandemic and, therefore, offer humanity a chance for a better future.

## Supplementary Information

**Additional file 1.**

## Data Availability

The data can be extracted from World Health Organization (WHO); World Bank (Worldwide Governance Indicators); Our World in Data/Worldometer; Oxford COVID-19 Government Response Tracker (Oxford University); European Quality of Government Index (EQI)—University of Gothenburg, Sweden; European Commission’s Standard Eurobarometer 94 “The EU and the pandemic coronavirus” (2021). The data used in the analysis will be available on request.

## Abbreviations

CC: Control of corruption
COVAX: COVID-19 Vaccines Global Access
CoVDP: COVID-19 Vaccine Delivery Partnership
EQI: European Quality of Government Index
EU: European Union
GDP: Gross domestic product
GE: Government effectiveness
GNI: Gross national income
PS: Political stability
RL: Rule of law
RQ: Regulatory quality
UNICEF: The United Nations Children’s Fund
US: United States
USAID: The United States Agency for International Development
V&A: Voice & accountability
WGI: Worldwide Governance Indicators
WHO: World Health Organization
