# Supplementary material for: COVID-19 vaccination and governance in the case of low, middle and high-income countries
Source: BMC Public Health. 2023 Jun 5;23:1073. doi: 10.1186/s12889-023-15975-3 (PMC10240451; doi:10.1186/s12889-023-15975-3)
Supplement: Supplementary file 1 — Additional file 1. [file 12889_2023_15975_MOESM1_ESM.docx]

Sample:

The sample consists of 170 countries, namely: Afghanistan (AFG); Albania (ALB); Algeria (DZA); Angola (AGO); Antigua and Barbuda (ATG); Argentina (ARG); Armenia (ARM); Aruba (ABW); Australia (AUS); Austria (AUT); Azerbaijan (AZE); Bahamas (BHS); Bahrain (BHR); Bangladesh (BGD); Barbados (BRB); Belarus (BLR); Belgium (BEL); Belize (BLZ); Benin (BEN); Bhutan (BTN); Bolivia (BOL); Bosnia and Herzegovina (BIH); Botswana (BWA); Brazil (BRA); Brunei (BRN); Bulgaria (BGR); Burkina Faso (BFA); Burundi (BDI); Cambodia (KHM); Cameroon (CMR); Canada (CAN); Cape Verde (CPV); Central African Republic (CAF); Chad (TCD); Chile (CHL); China (CHN); Colombia (COL); Congo (COG); Costa Rica (CRI); Cote d'Ivoire (CIV); Croatia (HRV); Cuba (CUB); Cyprus (CYP); Czechia (CZE); Democratic Republic of Congo (COD); Denmark (DNK); Djibouti (DJI); Dominica (DMA); Dominican Republic (DOM); Ecuador (ECU); Egypt (EGY); El Salvador (SLV); Estonia (EST); Ethiopia (ETH); Fiji (FJI); Finland (FIN); France (FRA); Gabon (GAB); Gambia (GMB); Georgia (GEO); Germany (DEU); Ghana (GHA); Greece (GRC); Grenada (GRD); Guatemala (GTM); Guinea-Bissau (GNB); Guyana (GUY); Haiti (HTI); Honduras (HND); Hong Kong (HKG); Hungary (HUN); Iceland (ISL); India (IND); Indonesia (IDN); Iran (IRN); Iraq (IRQ); Ireland (IRL); Israel (ISR); Italy (ITA); Jamaica (JAM); Japan (JPN); Jordan (JOR); Kazakhstan (KAZ); Kenya (KEN); Kosovo (KOS); Kuwait (KWT); Kyrgyzstan (KGZ); Laos (LAO); Latvia (LVA); Lebanon (LBN); Lesotho (LSO); Liberia (LBR); Libya (LBY); Lithuania (LTU); Luxembourg (LUX); Madagascar (MDG); Malawi (MWI); Malaysia (MYS); Maldives (MDV); Mali (MLI); Malta (MLT); Mauritania (MRT); Mauritius (MUS); Mexico (MEX); Moldova (MDA); Mongolia (MNG); Montenegro (MNE); Morocco (MAR); Mozambique (MOZ); Myanmar (MMR); Namibia (NAM); Nepal (NPL); Netherlands (NLD); New Zealand (NZL); Nicaragua (NIC); Niger (NER); Nigeria (NGA); North Macedonia (MKD); Norway (NOR); Oman (OMN); Pakistan (PAK); Panama (PAN); Papua New Guinea (PNG); Paraguay (PRY); Peru (PER); Philippines (PHL); Poland (POL); Portugal (PRT); Qatar (QAT); Romania (ROU); Russia (RUS); Rwanda (RWA); Saudi Arabia (SAU); Senegal (SEN); Serbia (SRB); Sierra Leone (SLE); Singapore (SGP); Slovakia (SVK); Slovenia (SVN); Somalia (SOM); South Africa (ZAF); South Korea (KOR); South Sudan (SSD); Spain (ESP); Sri Lanka (LKA); Sudan (SDN); Suriname (SUR); Sweden (SWE); Switzerland (CHE); Syria (SYR); Taiwan (TWN); Tajikistan (TJK); Tanzania (TZA); Thailand (THA) Timor (TLS); Togo (TGO); Trinidad and Tobago (TTO); Tunisia (TUN); Turkey (TUR); Uganda (UGA); Ukraine (UKR); United Arab Emirates (ARE); United Kingdom (GBR); United States (USA); Uruguay (URY); Uzbekistan (UZB); Venezuela (VEN); Vietnam (VNM); Yemen (YEM); Zambia (ZMB); Zimbabwe (ZWE).

**Appendix 1:** Ranking of countries in terms of COVID-19 vaccination and the six governance indicators (all sample, low, middle and high-income countries)

*All countries*


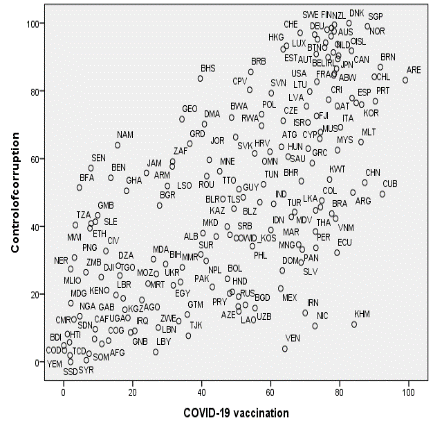

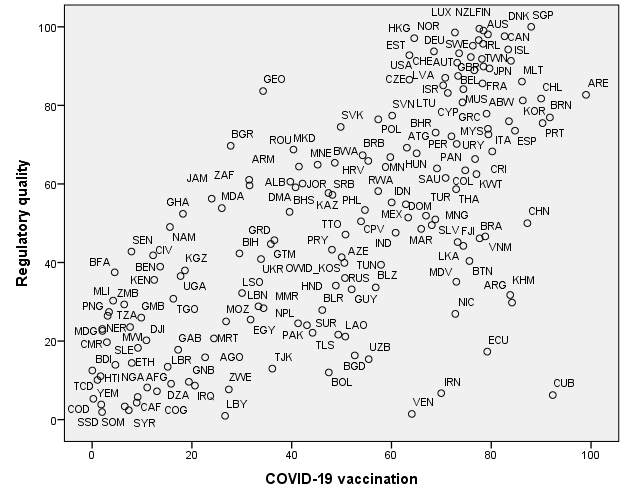

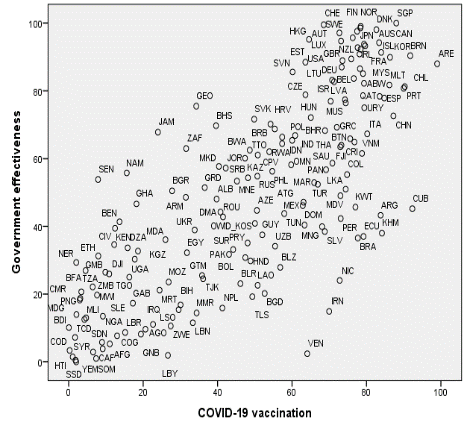


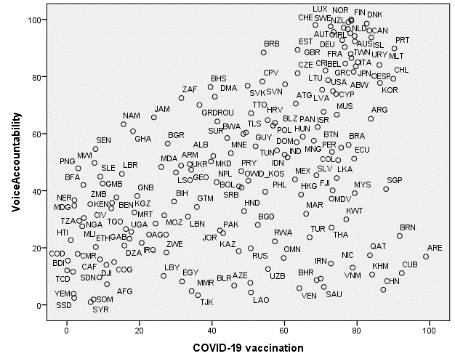

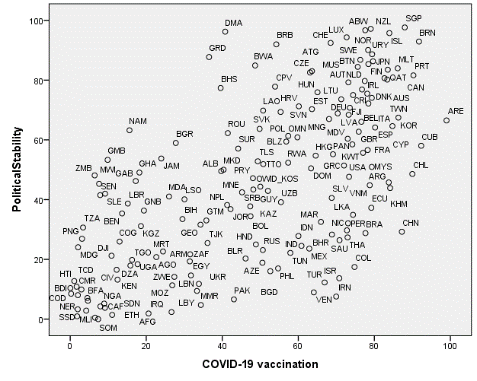

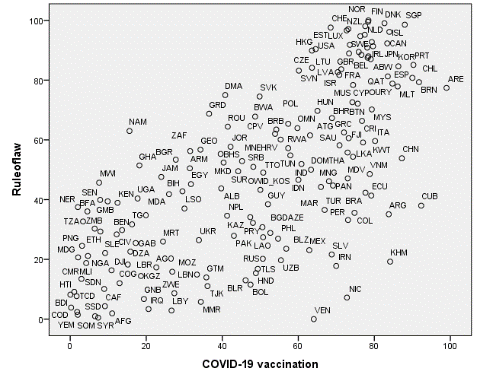


*Low-income countries*


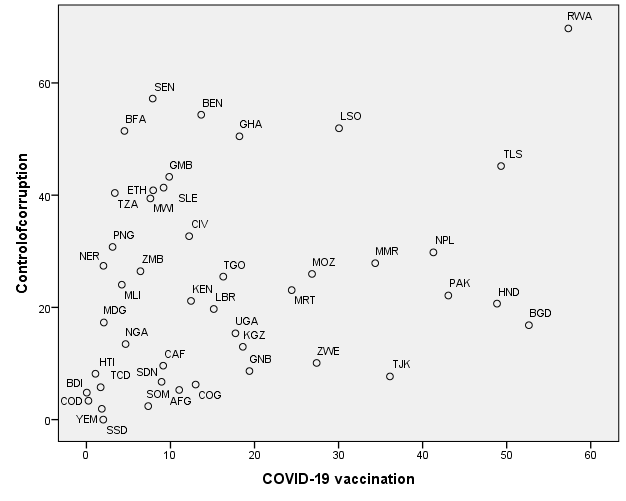

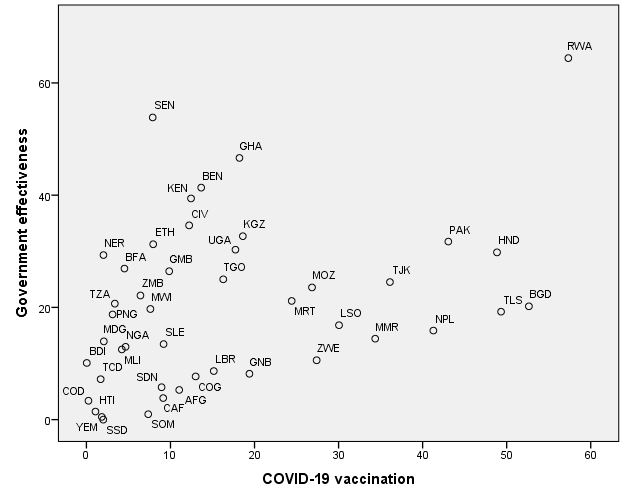

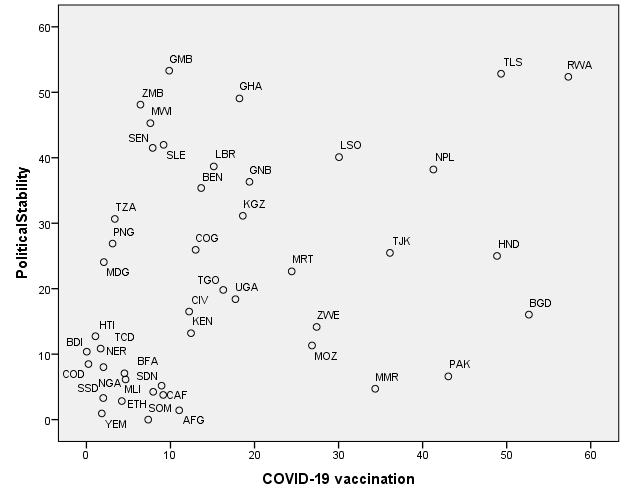


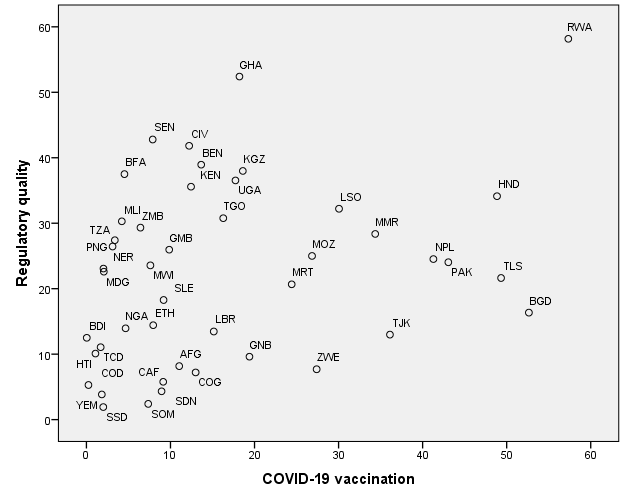

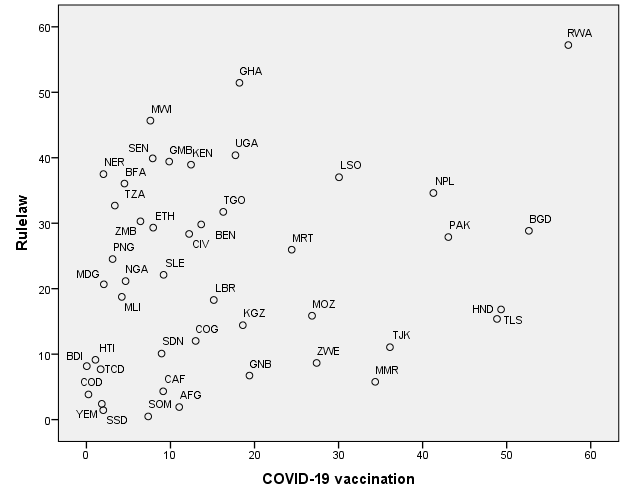

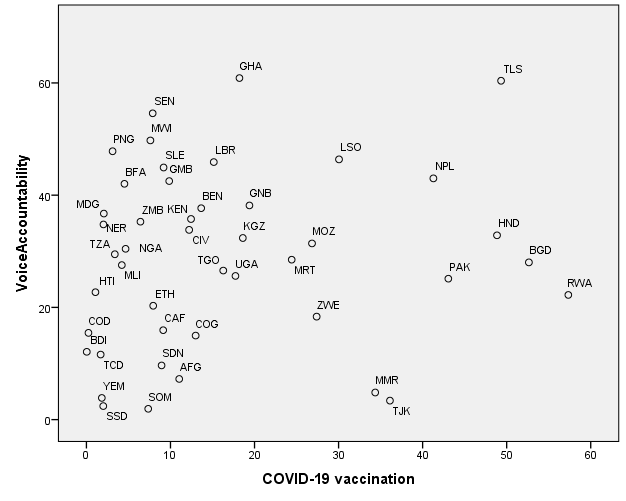


*Middle-income countries*


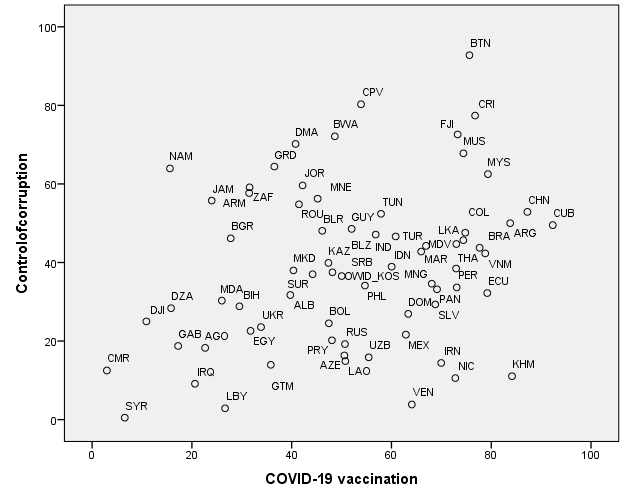

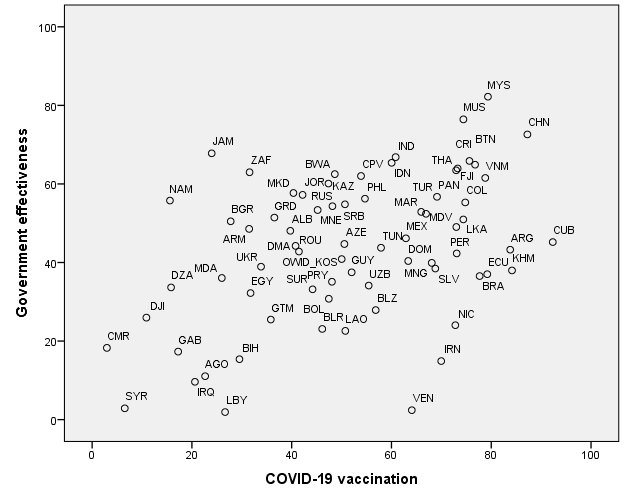

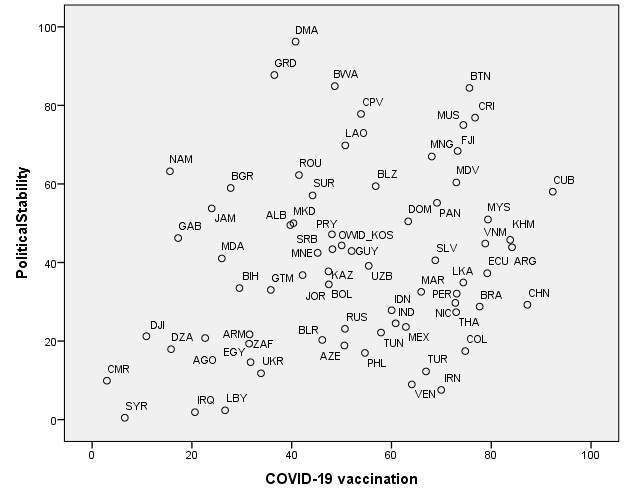


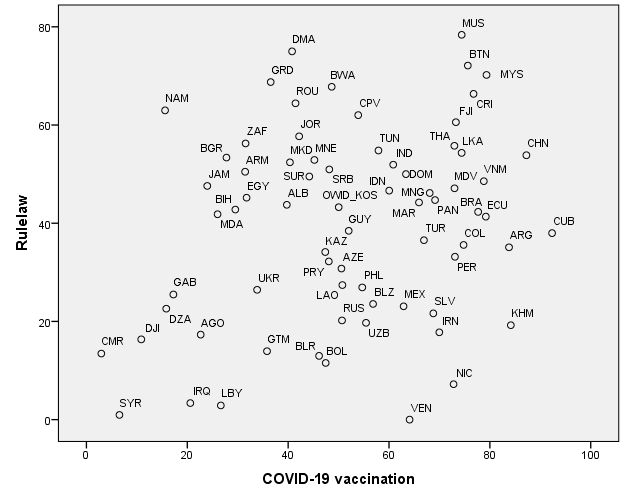

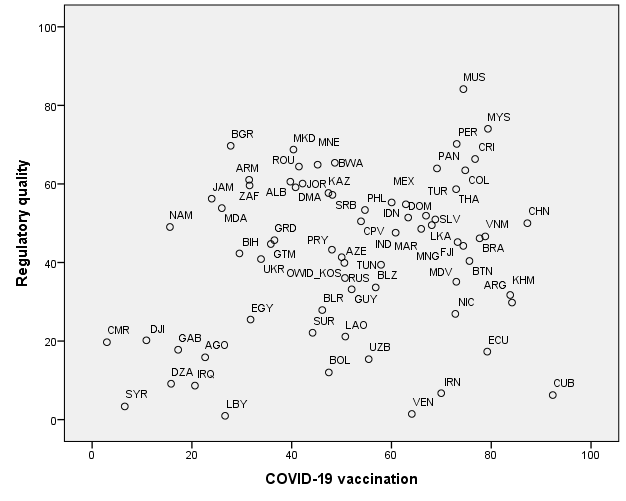

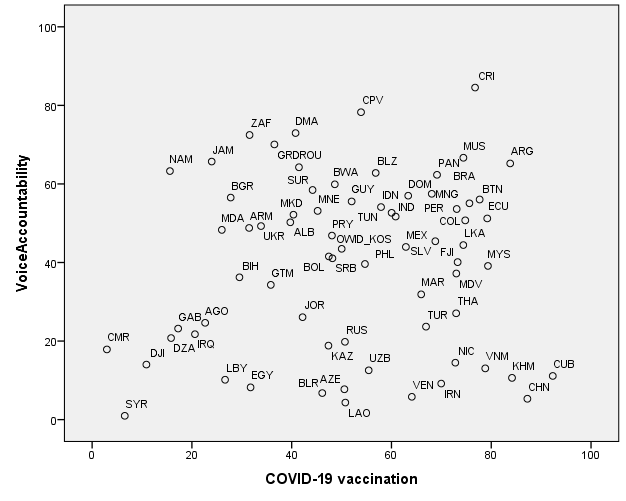


*High-income countries*


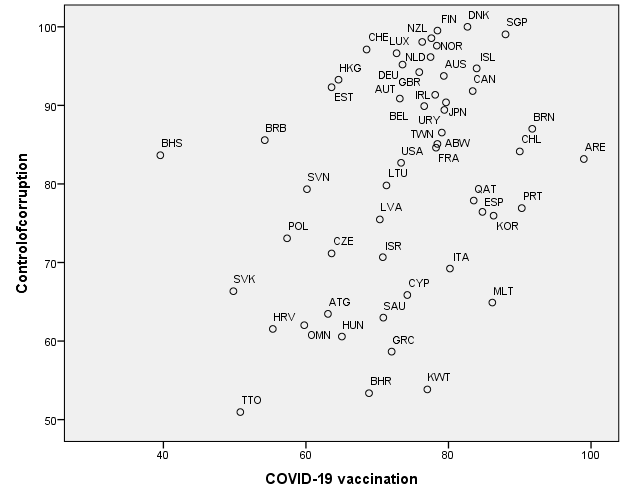

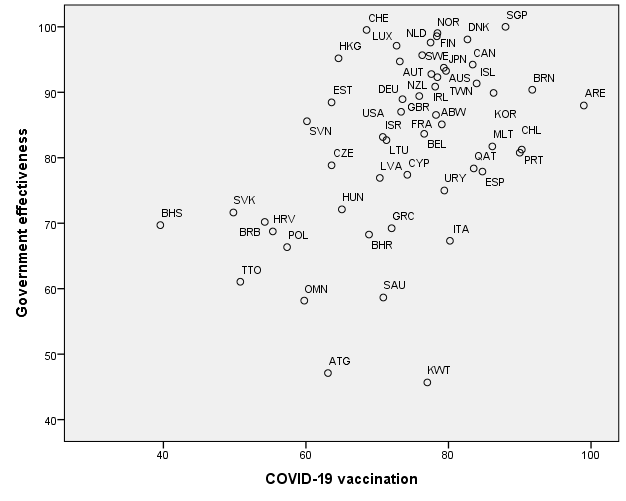

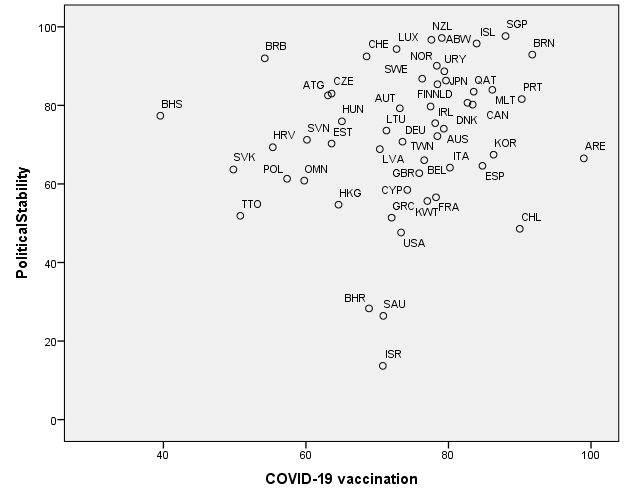


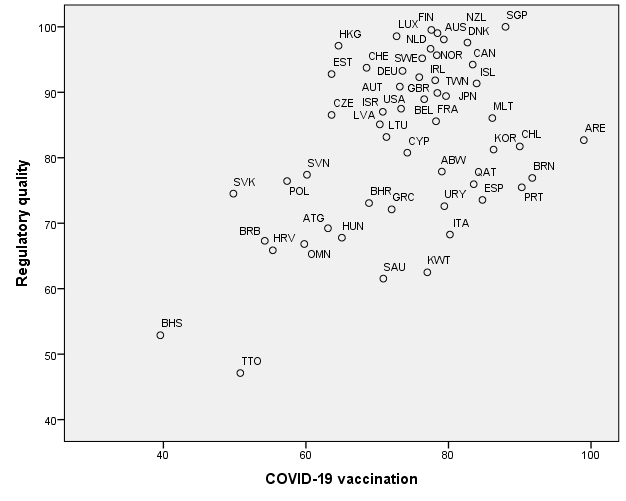

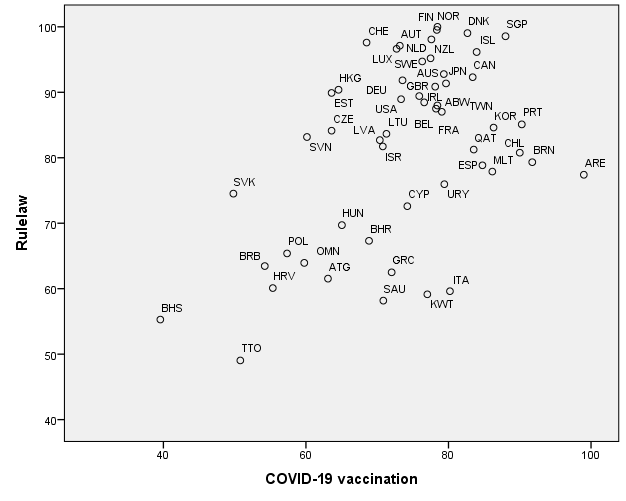

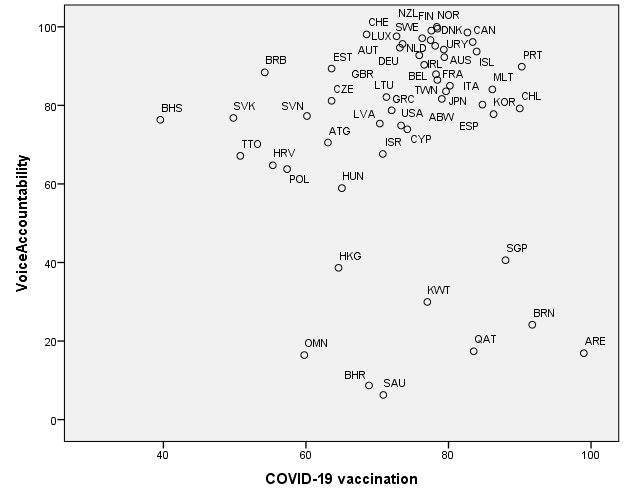


**Appendix 2:**

| COVID-19 vaccine booster doses administered per 100 people in high-, middle and low-income countries | |
| --- | --- |
| 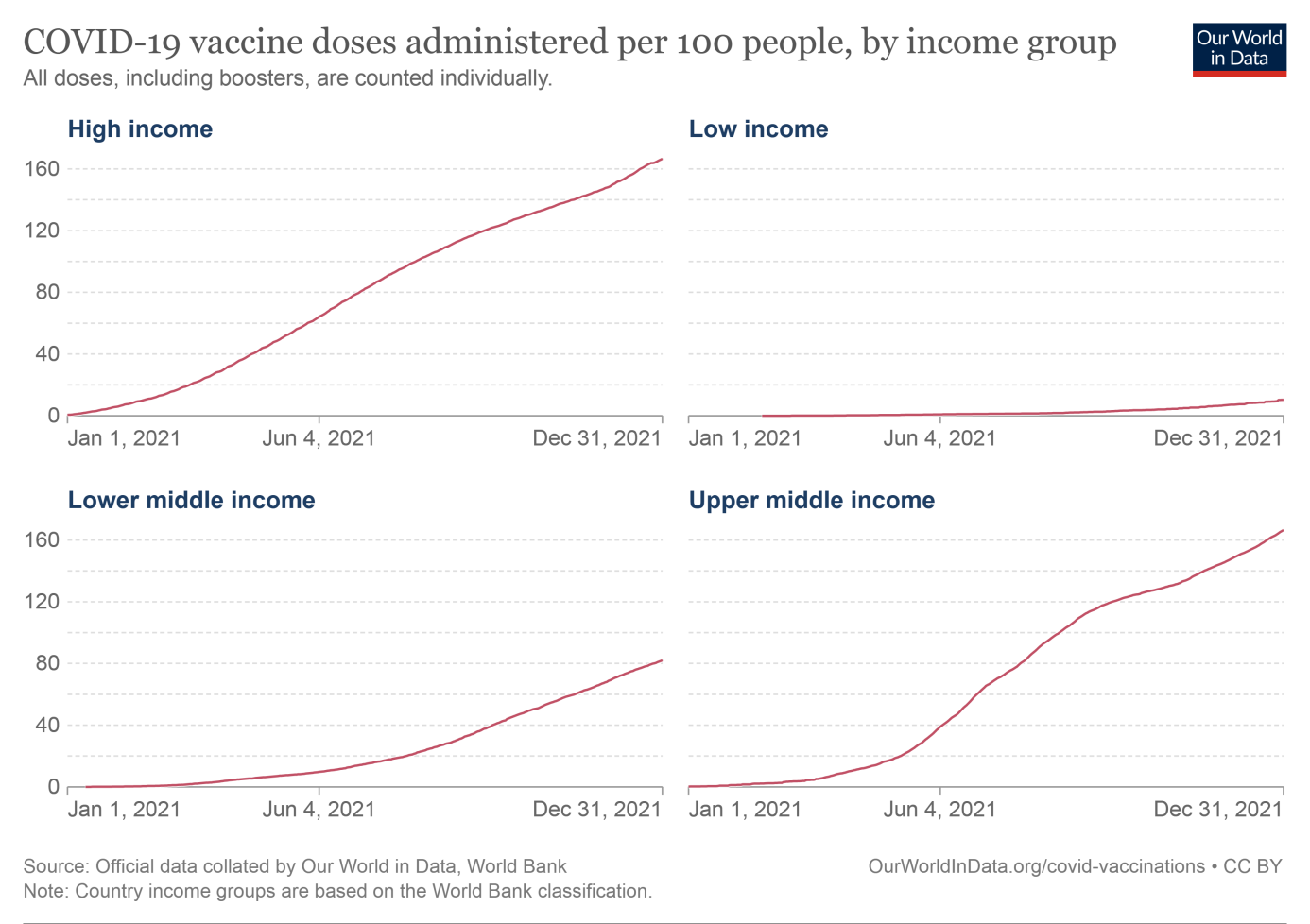 | 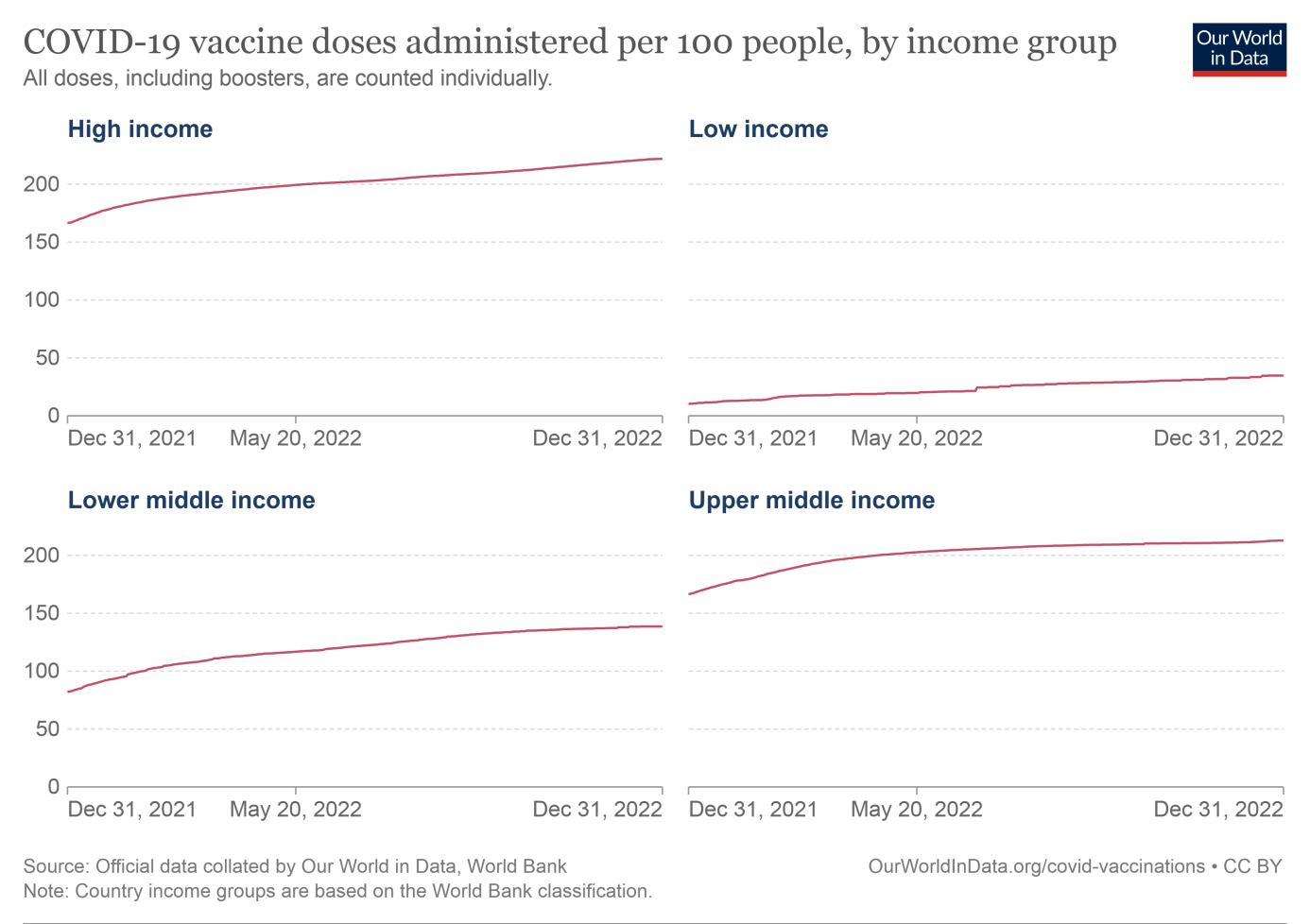 |
| Daily COVID-19 vaccine doses administered (7-day rolling average) | |
| 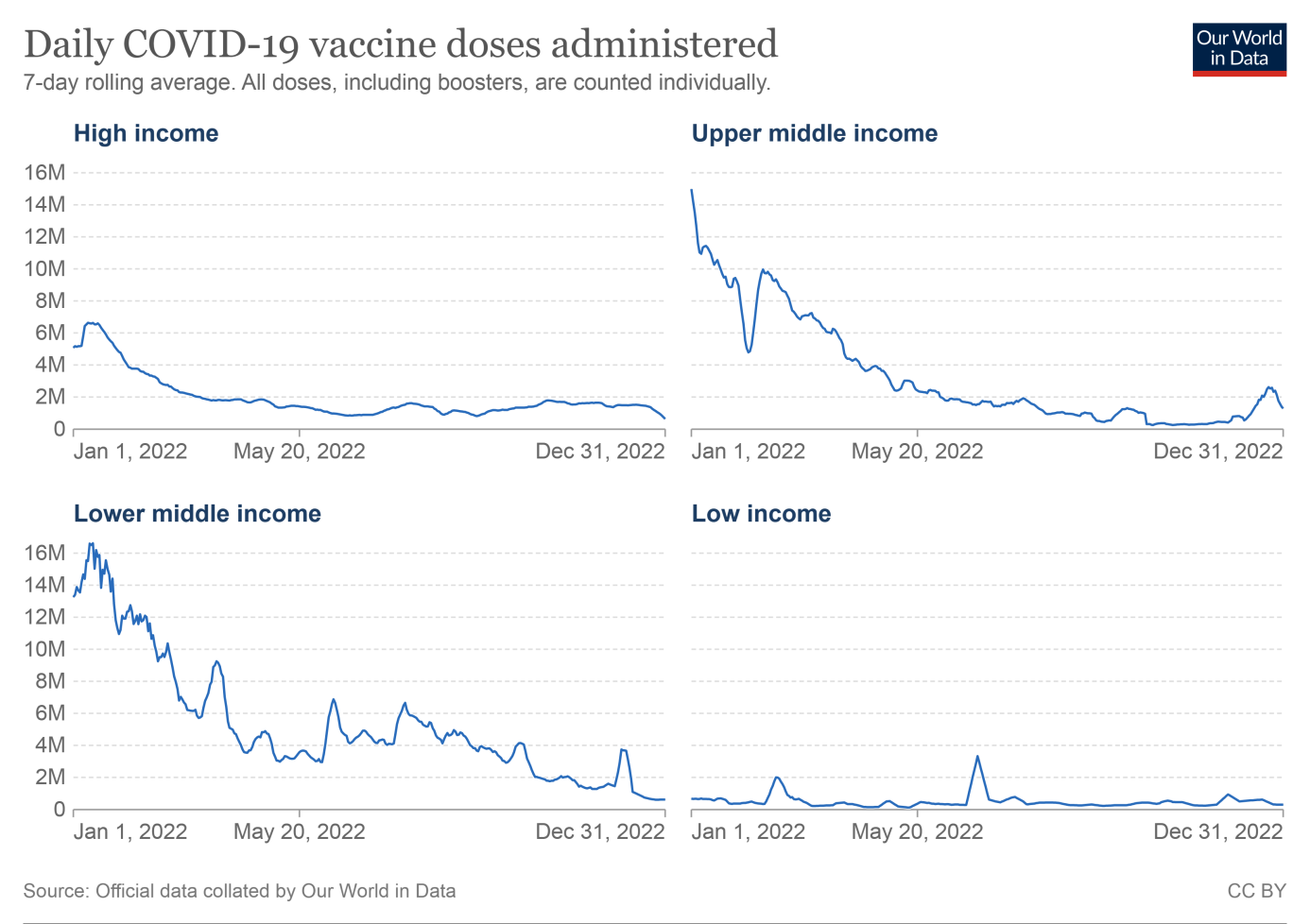 | |

*Source:* Our World in Data (World Bank), https://ourworldindata.org/covid-vaccinations
